# Improved fruit α‐tocopherol, carotenoid, squalene and phytosterol contents through manipulation of *Brassica juncea* 3‐HYDROXY‐3‐METHYLGLUTARYL‐COA SYNTHASE1 in transgenic tomato

**DOI:** 10.1111/pbi.12828

**Published:** 2017-10-17

**Authors:** Pan Liao, Xinjian Chen, Mingfu Wang, Thomas J. Bach, Mee‐Len Chye

**Affiliations:** ^1^ School of Biological Sciences The University of Hong Kong Pokfulam Hong Kong China; ^2^ Partner State Key Laboratory of Agrobiotechnology CUHK Shatin Hong Kong China; ^3^ Centre National de la Recherche Scientifique UPR 2357 Institut de Biologie Moléculaire des Plantes Strasbourg France

**Keywords:** isoprenoids, 3‐hydroxy‐3‐methylglutaryl‐coenzyme A synthase, mevalonate, phytosterols, squalene, tomato

## Abstract

3‐Hydroxy‐3‐methylglutaryl‐coenzyme A synthase (HMGS) in the mevalonate (MVA) pathway generates isoprenoids including phytosterols. Dietary phytosterols are important because they can lower blood cholesterol levels. Previously, the overexpression of *Brassica juncea* wild‐type (wt) and mutant (S359A) BjHMGS1 in *Arabidopsis* up‐regulated several genes in sterol biosynthesis and increased sterol content. Recombinant S359A had earlier displayed a 10‐fold higher *in vitro* enzyme activity. Furthermore, tobacco HMGS overexpressors (OEs) exhibited improved sterol content, plant growth and seed yield. Increased growth and seed yield in tobacco OE‐S359A over OE‐wtBjHMGS1 coincided with elevations in *NtSQS* expression and sterol content. Herein, the overexpression of wt and mutant (S359A) BjHMGS1 in a crop plant, tomato (*Solanum lycopersicum*), caused an accumulation of MVA‐derived squalene and phytosterols, as well as methylerythritol phosphate (MEP)‐derived α‐tocopherol (vitamin E) and carotenoids, which are important to human health as antioxidants. In tomato HMGS‐OE seedlings, genes associated with the biosyntheses of C10, C15 and C20 universal precursors of isoprenoids, phytosterols, brassinosteroids, dolichols, methylerythritol phosphate, carotenoid and vitamin E were up‐regulated. In OE‐S359A tomato fruits, increased squalene and phytosterol contents over OE‐wtBjHMGS1 were attributed to heightened *SlHMGR2*,* SlFPS1*,* SlSQS* and *SlCYP710A11* expression. In both tomato OE‐wtBjHMGS1 and OE‐S359A fruits, the up‐regulation of *SlGPS* and *SlGGPPS1* in the MEP pathway that led to α‐tocopherol and carotenoid accumulation indicated cross‐talk between the MVA and MEP pathways. Taken together, the manipulation of BjHMGS1 represents a promising strategy to simultaneously elevate health‐promoting squalene, phytosterols, α‐tocopherol and carotenoids in tomato, an edible fruit.

## Introduction

In plant cells, isoprenoids including phytosterols, sesquiterpenes, monoterpenes, cytokinins (CKs), carotenoids, vitamin E, dolichol and brassinosteroids (BRs) are generated via the mevalonate (MVA) pathway and/or the methylerythritol phosphate (MEP) pathway (Akhtar *et al*., 2013; Besser *et al*., 2009; Enfissi *et al*., 2010; Rodríguez‐Concepción and Gruissem, 1999; Sallaud *et al*., 2009). Isopentenyl diphosphate isomerase (IPI) catalyses the interconversion of isopentenyl diphosphate (IPP) and its allyl isomer dimethylallyl diphosphate (DMAPP) and provides the first key intermediate for all kinds of isoprenoids including sterols (Bach, 1995; Hemmerlin *et al*., 2012; Rohmer, 1999; Sacchettini and Poulter, 1997). IPP is involved in cross‐talk between the cytosolic MVA pathway and the plastidial MEP pathway (Hemmerlin *et al*., 2003; Laule *et al*., 2003). Some cross‐regulations between them are also known to occur (Hemmerlin *et al*., 2012 and references therein; Huchelmann *et al*., 2014; Liao *et al*., 2016 and references therein).

The MEP pathway generates monoterpenes, diterpenes, carotenoids, tocopherols and class II sesquiterpenes. Geranyl diphosphate synthase (GPS) is responsible for the biosynthesis of monoterpene precursors, GPP and some FPPs (van Schie *et al*., 2007). Subsequently, monoterpene synthases (MTS) act to produce monoterpenes (Besser *et al*., 2009). Geranylgeranyl diphosphate synthase (GGPPS) catalyses the formation of 20‐carbon geranylgeranyl diphosphate (GGPP), which is the universal precursor of carotenoids, diterpenes, gibberellins (GAs) and vitamin E (Lichtenthaler, 1999; Rohmer, 1999). GGPP reductase (GGPPR) and γ‐methyl tocopherol transferase (GMTT) are two enzymes responsible for tocopherol biosynthesis in plants (Camara and d'Harlingue, 1985; Enfissi *et al*., 2010). Of the four naturally occurring species of tocopherols (vitamin E), α‐tocopherol is the most important one to human health as it has higher antioxidant activity than β‐, γ‐ or δ‐tocopherols (Azzi, 2007; DellaPenna, 2005; Shintani and DellaPenna, 1998).

3‐Hydroxy‐3‐methylglutaryl‐coenzyme A synthase (HMGS), which represents the second enzyme in the MVA pathway, can be genetically engineered to overaccumulate phytosterol content (Lange *et al*., 2015; Liao *et al*., 2014b; Wang *et al*., 2012). The importance of *Arabidopsis thaliana* HMGS in sterol biosynthesis, pollen grain fertility and seed yield has been demonstrated (Bhangu‐Uhlmann, 2011; Ishiguro *et al*., 2010; Lange *et al*., 2015; Liao *et al*., 2014a). In *Brassica juncea*, four isogenes encode HMGS (Alex *et al*., 2000). A mutant recombinant BjHMGS1 (S359A) was reported to show a 10‐fold increase in enzyme activity *in vitro* (Nagegowda *et al*., 2004). Interestingly, for the *Enterococcus faecalis* HMGS mutant, A110G, the reaction rate was elevated 140‐fold, because the amino acid substitution in A110G caused repositioning of the hydroxyl group in Ser308, the equivalent to *B. juncea* HMGS Ser359 (Steussy *et al*., 2006). It was proposed that *B. juncea* HMGS S359A has a shorter side chain, bringing the backbone of S359A closer to the catalytic loop, expediting the reaction (Steussy *et al*., 2006). Transgenic Arabidopsis HMGS‐OEs driven by the CaMV *35S* promoter displayed induced expression of *A. thaliana 3‐HYDROXY‐3‐METHYLGLUTARYL‐COENZYME A REDUCTASE* (*AtHMGR)*,* STEROL METHYLTRANSFERASE2* (*AtSMT2)*, Δ^24^ *STEROL REDUCTASE (AtDWF1)*,* STEROL C‐22 DESATURASE (AtCYP710A1)* and *BRASSINOSTEROID‐6‐OXIDASE2 (AtBR6OX2)* (Wang *et al*., 2012). Phytosterol content was increased in Arabidopsis OE‐wtBjHMGS1 (11.3% and 13.6% enhancements in total seedling and leaf sterol content, respectively) and OE‐S359A (26.8% and 22.3% elevations in total seedling and leaf sterol content, respectively) (Wang *et al*., 2012). Tobacco (*Nicotiana tabacum*) HMGS‐OEs driven by the CaMV *35S* promoter showed an up‐regulation of *NtHMGR1*,* NtIPI2*,* SQUALENE SYNTHASE* (*NtSQS)*,* NtSMT1‐2*,* NtSMT2‐1*,* NtSMT2‐2*,* CYTOCHROME P450 MONOOXYGENASE* (*NtCYP85A1)* and *NtGGPPS2*, but down‐regulation of *NtIPI1*,* NtGGPPS1*,* NtGGPPS3* and *NtGGPPS4* (Liao *et al*., 2014b). In addition, the expression of *NtSQS*,* NtHMGR1*,* NtSMT2‐1* and *NtCYP85A1* in tobacco S359A overexpressors (OE‐S359A) was even higher than in the wild‐type (wt) BjHMGS1 overexpressors (OE‐wtBjHMGS1) (Liao *et al*., 2014b). Phytosterol content was also promoted in tobacco OE‐wtBjHMGS1 (4.6% and 12.1% in total seedling and leaf sterol content, respectively) and OE‐S359A (22.9% and 18.7% in total seedling and leaf sterol content, respectively), with OE‐S359A seedlings showing higher phytosterol content than OE‐wtBjHMGS1 (Liao *et al*., 2014b). Furthermore, tobacco HMGS‐OEs displayed enhanced plant growth, pod size and seed yield, with OE‐S359A exhibiting a greater effect than OE‐wtBjHMGS1 (Liao *et al*., 2014b).

Given that dietary phytosterols have been reported to lower blood cholesterol levels and might thereby reduce the risk of heart disease (Bradford and Awad, 2007; Moreau *et al*., 2002; Woyengo *et al*., 2009), it would be strategic to genetically manipulate HMGS in an edible fruit such as tomato. The metabolic engineering of the tomato MVA pathway using HMGR had caused a 2.4‐fold increase in phytosterol content in mature transgenic T_0_ tomato fruits overexpressing Arabidopsis HMGR1 (HMGR1‐OE) (Enfissi *et al*., 2005). However, in the mature fruits of the homozygous T_2_ tomato HMGR1‐OE, total HMGR activity was not enhanced and only few specific phytosterols were elevated (Enfissi *et al*., 2005), indicating that phytosterol increase in tomato HMGR1‐OEs was not stably inherited (Enfissi *et al*., 2005). Furthermore, HMGR is known to be subject to regulation at transcriptional, post‐transcriptional, translational and post‐translational levels (Bach, 1986; Hemmerlin, 2013; Wong *et al*., 1982).

Given the positive effects of BjHMGS1 in elevating phytosterols in HMGS‐OEs of model plants such as Arabidopsis and tobacco, and the improved effect of OE‐S359A in plant growth besides sterol content, the application of S359A should be extended to benefit food crops. Tomato (*Solanum lycopersicum*) was selected in this study because it is a popular fruit, eaten raw and from which juice is easily extracted for human consumption. Furthermore, the technology for tomato transformation is available (Mathews *et al*., 2003). To comprehensively investigate the effects of HMGS in regulating isoprenoid biosynthesis in tomato, the expression of genes in tomato HMGS‐OEs involved in the biosyntheses of MVA, C10, C15 and C20 universal precursors of isoprenoids, phytosterols, BR, CKs, dolichols, monoterpenes, sesquiterpenes, MEP, carotenoids and vitamin E was analysed by quantitative reverse transcription PCR (qRT‐PCR). The effect of HMGS overexpression on the metabolic flux was subsequently investigated by gas chromatography–mass spectrometry (GC‐MS) and high‐performance liquid chromatography (HPLC). It is interesting to note that the overexpression of BjHMGS1 in tomato up‐regulated *SlGPS* and *SlGGPPS*, causing a significant elevation in fruit α‐tocopherol and carotenoids, products of the MEP pathway, besides those of the MVA pathway such as squalene and phytosterols. Overall, this study demonstrates the potential of BjHMGS1 in simultaneously promoting vitamin E, carotenoid, squalene and phytosterol production in edible plants that would benefit human health.

## Results

### Tomato HMGS‐OE plants showed increased growth

**Figure 1:**
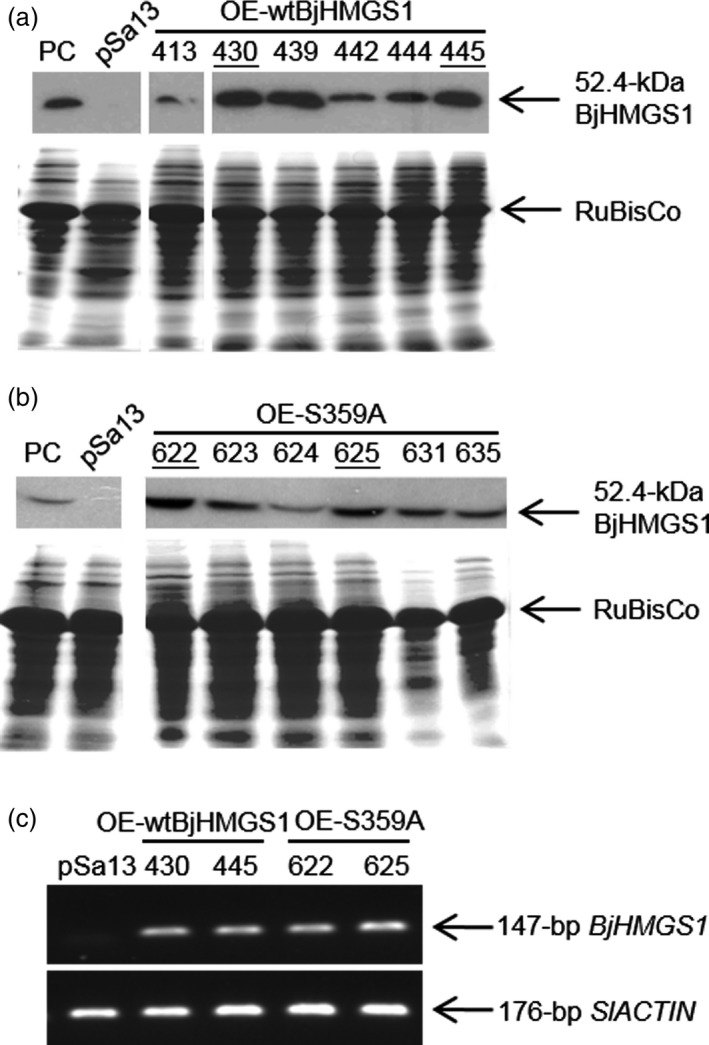
Characterization of representative transgenic tomato HMGS‐OEs. (a, b) Western blot analysis using antibodies against BjHMGS1 to verify the expression of BjHMGS1 in tomato leaves of 5‐week‐old representative wild‐type HMGS‐OEs (OE‐wtBjHMGS1) in (a) and mutant HMGS‐OEs (OE‐S359A) in (b). The cross‐reacting HMGS band is indicated by an arrowhead. Putative tomato HMGS‐OEs were designated as OE‐wtBjHMGS1 lines (413, 430, 439, 442, 444 and 445) in (a) and OE‐S359A lines (622, 623, 624, 625, 631 and 635) in (b). PC, positive control (tobacco BjHMGS1 OE line 402 as reported in Liao *et al*., 2014b); pSa13, vector (pSa13)‐transformed tomato. Bottom, Coomassie Blue‐stained gel of 20 μg total protein in each well. Two independent lines from each construct selected for further tests are underlined. White lines have been inserted between lanes that have been spliced together from the same original gel/blot. (c) Semiquantitative RT‐PCR analysis on representative transgenic tomato plants. *BjHMGS1*‐specific primers (ML1666 and ML1667) and tomato *ACTIN* (*SlACTIN*)‐specific primers (ML1688 and ML1689) were used. The PCR bands of 147‐bp *BjHMGS1* and 176‐bp *SlACTIN* are indicated. pSa13, vector (pSa13)‐transformed control.

**Figure 2:**
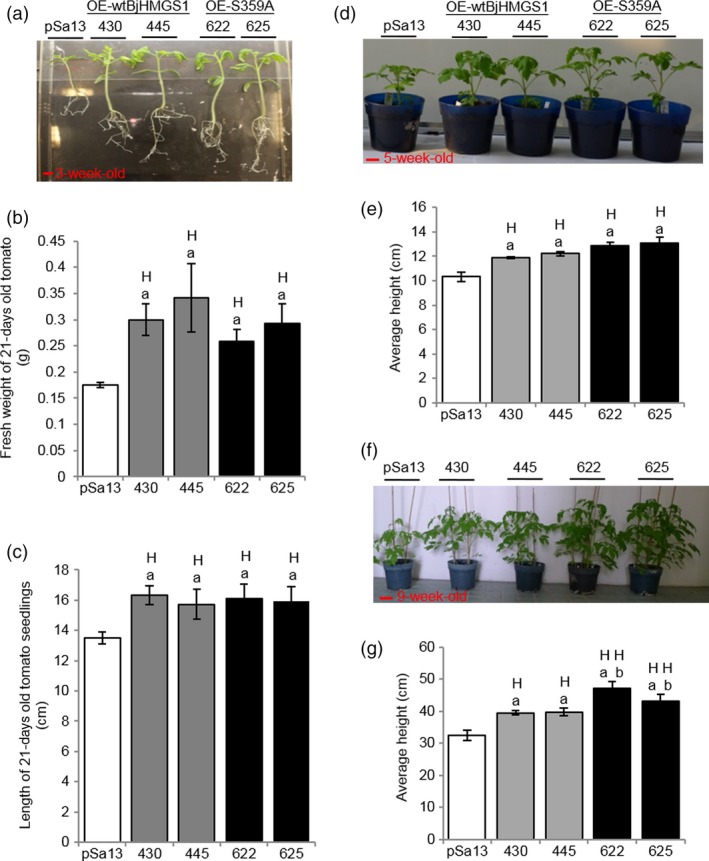
Comparison in growth between 3‐, 5‐, 9‐week‐old tomato HMGS‐OE seedlings/plants and vector‐transformed control. Two independent lines each of OE‐wtBjHMGS1 (430 and 445) and OE‐S359A (622 and 625) lines were compared to the vector‐transformed control (pSa13). (a) Representative MS plate‐grown seedlings photographed 3 weeks after germination. Bar = 1 cm. (b) Statistical analysis on fresh weight of 3‐week‐old tomato seedlings. (c) Statistical analysis on length of 3‐week‐old tomato seedlings. (d) Representative greenhouse‐grown plants photographed 5 weeks after germination. Bar = 3 cm. (e) Statistical analysis on height of 5‐week‐old tomato plants. (f) Representative greenhouse‐grown plants photographed 9 weeks after germination. Bar = 6 cm. (g) Statistical analysis on height of 9‐week‐old tomato plants. Values are mean ± SD (*n* = 30); bars are SD; H, value higher than the control; L, value lower than the control. ‘a’ indicates significant difference between HMGS‐OE and the vector (pSa13)‐transformed control; ‘b’ indicates significant difference between OE‐wtBjHMGS1 and OE‐S359A (*P *<* *0.01, Student's *t*‐test).

To check whether BjHMGS1 overexpression in verified tomato lines (Figures 1, S1 and S2) caused enhanced growth as previously observed in tobacco HMGS‐OEs (Liao *et al*., 2014b), the length and fresh weight of 3‐week‐old (Figure 2a–c) and 5‐week‐old (Figure 2d and e) seedlings and the height of 9‐week‐old tomato plants (Figure 2f and g) were compared amongst OE‐wtBjHMGS1, OE‐S359A and the vector‐transformed lines. Three‐week‐old OE‐wtBjHMGS1 and OE‐S359A tomato seedlings had a higher fresh weight (83% and 57%, respectively, in Figure 2a and b) and longer seedling length (16% and 19%, respectively, in Figure 2a and c) than the control, but there was no significant difference in growth between 3‐week‐old OE‐wtBjHMGS1 and OE‐S359A seedlings (Figure 2b and c). Correspondingly, 5‐week‐old transgenic OE‐wtBjHMGS1 and OE‐S359A tomato plants displayed significant increases (17% and 26%, respectively) in height over the control (Figure 2d and e). Consistently, 9‐week‐old HMGS‐OEs (OE‐wtBjHMGS1 and OE‐S359A) grew better than the control (Figure 2f); OE‐wtBjHMGS1 displayed a 22% increase in height over the control, while OE‐S359A displayed an even higher (39%) increase as shown in Figure 2g.

### Effect of HMGS overexpression on MVA‐related gene expression in tomato HMGS‐OE seedlings

**Figure 3:**
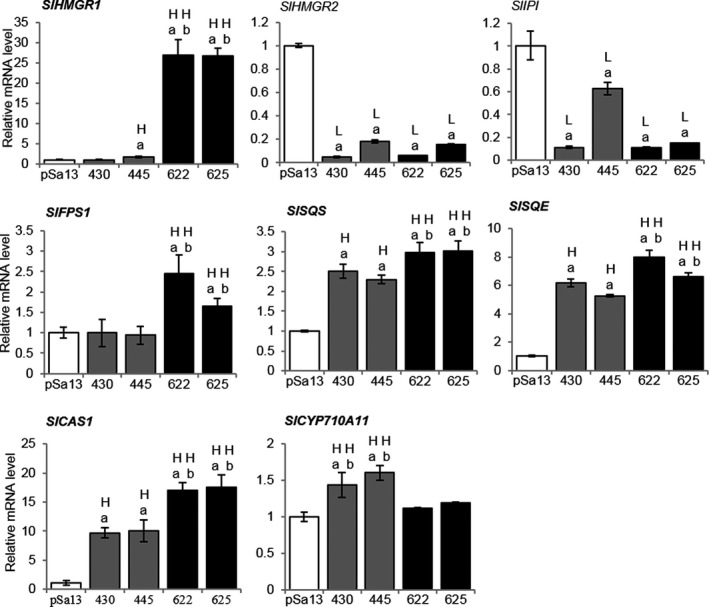
QRT‐PCR analysis on the expression of MVA‐ and phytosterol‐related genes in tomato HMGS‐OE seedlings. Total RNA was extracted from 3‐week‐old tomato seedlings of the vector (pSa13)‐transformed control, OE‐wtBjHMGS1 lines (430 and 445) and OE‐S359A lines (622 and 625). H, value higher than the control; L, value lower than the control. Values are means ± SD (*n* = 3). ‘a’ indicates significant difference between HMGS‐OE and the vector (pSa13)‐transformed control; ‘b’ indicates significant difference between OE‐wtBjHMGS1 and OE‐S359A (*P *<* *0.05, Student's *t*‐test).

Quantitative reverse transcription PCR was performed to test the effect of *BjHMGS1* overexpression on the expression of genes downstream of *HMGS*, as well as of genes responsible for the biosyntheses of C10, C15 and C20 universal precursors of isoprenoids, and of sesquiterpenes in tomato HMGS‐OE seedlings. In OE‐wtBjHMGS1, *SlSQS*,* SQUALENE EPOXIDASE (SlSQE)* and *CYCLOARTENOL SYNTHASE1* (*SlCAS1*) mRNAs were induced (Figure 3). In OE‐S359A, additional genes including *SlHMGR1*,* FARNESYL DIPHOSPHATE SYNTHASE1 (SlFPS1)*,* SlSQS*,* SlSQE*,* SlCAS1* and sesquiterpene‐related genes (*SlSSTLE1* and *SlSSTLH3*) were up‐regulated (Figures 3 and S3). Greater expression of *SlHMGR1*,* SlFPS1*,* SlSQS*,* SlSQE*,* SlCAS1*,* SlSSTLE1* and *SlSSTLH3* in OE‐S359A than that of OE‐wtBjHMGS1 seedlings (Figures 3 and S3) corresponded to significant growth enhancement in 9‐week‐old OE‐S359A plants (Figure 2f and g). Furthermore, the expression of BR‐related genes (*SlCYP85A1* and *SlCYP85A3*), CK‐related genes that encode type‐A response regulator proteins (*SlTRR3/4*,* SlTRR8/9a*,* SlTRR8/9b* and *SlTRR16/17*) (Shani *et al*., 2010) and dolichol‐related *cis‐PRENYLTRANSFERASE (SlCPT3)* was significantly elevated in both OE‐wtBjHMGS1 and OE‐S359A over the vector control (Figure S4a–c), coinciding with increased growth in tomato HMGS‐OEs (Figure 2). *SlCYP85A3* and *SlCPT3* expression in OE‐S359A was much higher than in OE‐wtBjHMGS1 (Figure S4a and c), corresponding well to better growth enhancement in 9‐week‐old OE‐S359A plants (Figure 2f and g). However, *SlHMGR2* and *SlIPI* expression significantly decreased in all OE‐wtBjHMGS1 and OE‐S359A lines (Figure 3).

### HMGS overexpression affected MEP‐related genes in tomato seedlings

**Figure 4:**
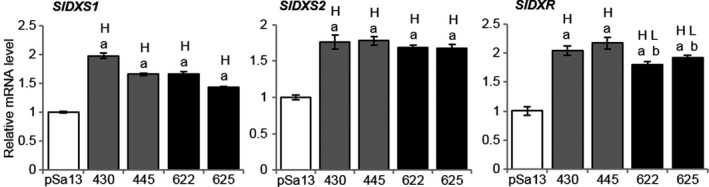
QRT‐PCR analysis on the expression of MEP pathway‐related genes in tomato HMGS‐OE seedlings. Total RNA was extracted from 3‐week‐old tomato seedlings of the vector (pSa13)‐transformed control, OE‐wtBjHMGS1 lines (430 and 445) and OE‐S359A lines (622 and 625). H, value higher than the control; L, value lower than the control. Values are means ± SD (*n* = 3). ‘a’ indicates significant difference between HMGS‐OE and the vector (pSa13)‐transformed control; ‘b’ indicates significant difference between OE‐wtBjHMGS1 and OE‐S359A (*P *<* *0.05, Student's *t*‐test).

MEP‐related genes [*1‐DEOXY‐D‐XYLULOSE 5‐PHOSPHATE SYNTHASE1 (SlDXS1)*,* SlDXS2* and *1‐DEOXY‐D‐XYLULOSE 5‐PHOSPHATE REDUCTOISOMERASE (SlDXR)*] were slightly induced in OE‐wtBjHMGS1 and OE‐S359A in comparison with the vector control (Figure 4). The expression of genes (*SlCPT1*,* SlCPT2*,* SlCPT6*,* SlGPS*,* SlGPPS‐SSU‐II*,* SlGGPPS1* and *SlGGPPS2*) leading to the biosynthesis of plastidial C10 and C20 universal precursors of isoprenoids was higher in OE‐wtBjHMGS1 and OE‐S359A than in the control (Figure S5a). *SlCPT1* and *SlCPT2* expression in OE‐S359A was much higher than in OE‐wtBjHMGS1 (Figure S5a), corresponding to significant growth enhancement in 9‐week‐old OE‐S359A plants (Figure 2f and g).

It is interesting to note that carotenoid‐related genes [(*PHYTOENE SYNTHASE (SlPSY‐1)*,* SlPSY‐2*, ζ‐CAROTENE DESATURASE *(SlZDS)*,* CAROTENE ISOMERASE (SlCRTISO)*, β*‐LYCOPENE CYCLASE (SlLCY‐B)*, β*‐LYCOPENE CYCLASE (SlCYC‐B)* and ε*‐LYCOPENE CYCLASE (SlLCY‐E)* except *PHYTOENE DESATURASE* (*SlPDS)*] and vitamin E‐related genes (*SlGGPPR* and *SlGMTT*) were up‐regulated in tomato HMGS‐OE seedlings (Figure S6a and b). Furthermore, *SlPDS*,* SlCRTISO* and *SlCYC‐B* expression in OE‐S359A was much higher than in OE‐wtBjHMGS1 (Figure S6a). However, there was no difference in monoterpene‐related *SlMTS1* expression amongst the vector control, OE‐wtBjHMGS1 and OE‐S359A (Figure S5b).

### Tomato HMGS‐OE fruits accumulated MVA‐derived squalene, sterol‐related intermediates, sterols and MEP‐derived vitamin E and carotenoids

Given the induced expression of isoprenoid‐, vitamin E‐ and carotenoid‐related genes in tomato HMGS‐OE seedlings and tomato fruit is edible, analysis on tomato HMGS‐OE fruits would inform on feasibility in the accumulation of health‐promoting end products from the MVA and MEP pathways such as phytosterols and related intermediates, vitamin E and carotenoids.

**Figure 5:**
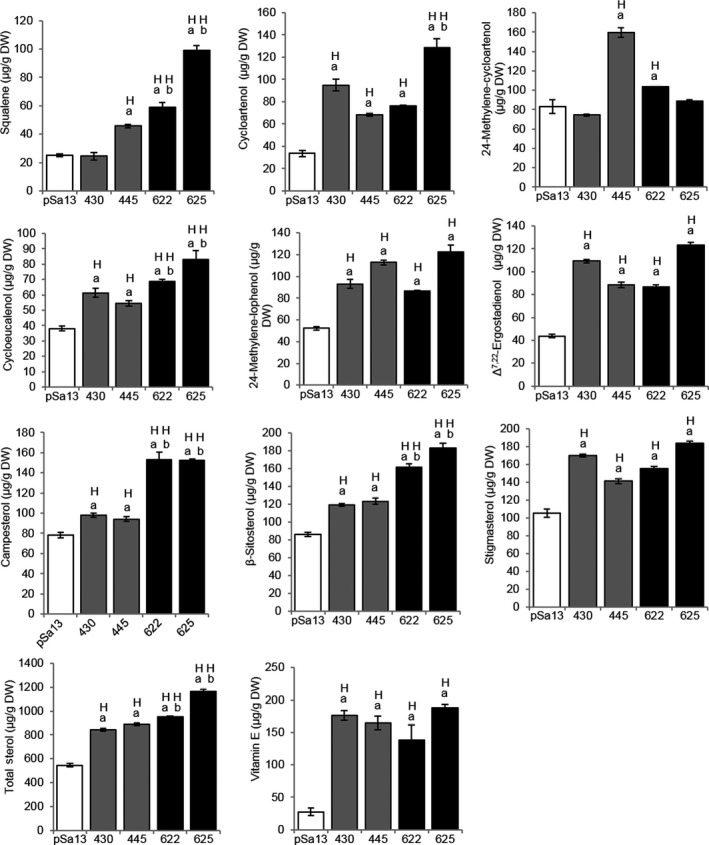
GC‐MS analysis of squalene, sterol‐related intermediate, sterol and vitamin E contents [μg/g dry weight (DW)] in 57 DAP mature tomato HMGS‐OE fruits. Lipids were extracted from the vector‐transformed control (pSa13), two independent lines of OE‐wtBjHMGS1 (430 and 445) and two independent lines of OE‐S359A (622 and 625). H, value higher than the control (*P *<* *0.01, Student's *t*‐test); L, value lower than the control (*P *<* *0.01, Student's *t*‐test). Values are mean ± SD (*n* = 8); bars are SD; ‘a’ indicates significant difference between HMGS‐OE and the vector (pSa13)‐transformed control; ‘b’ indicates significant difference between OE‐wtBjHMGS1 and OE‐S359A (*P *<* *0.01, Student's *t*‐test).

When the contents of major phytosterols (campesterol, β‐sitosterol and stigmasterol) and related intermediates (squalene, cycloartenol, 24‐methylene‐cycloartanol, cycloeucalenol, 24‐methylene‐lophenol and Δ^7,22^‐ergostadienol) in tomato HMGS‐OE fruits were analysed by GC‐MS, the results showed an increase in OE‐BjHMGS1 and OE‐S359A over the vector control, with the exception of squalene (in OE‐BjHMGS1 line 430) and 24‐methylene‐cycloartanol (in OE‐BjHMGS1 line 430 and OE‐S359A line 625) (Figure 5). Furthermore, squalene, cycloeucalenol, campesterol and β‐sitosterol contents in OE‐S359A were significantly higher than in OE‐BjHMGS1 (Figure 5).

In particular, such elevation was evident in squalene (average elevation of 39.2%), cycloartenol (140%), 24‐methylene‐cycloartanol (40.8%), cycloeucalenol (52.0%), 24‐methylene‐lophenol (96.8%), campesterol (22.9%), β‐sitosterol (40.1%), stigmasterol (47.7%), Δ^7,22^‐ergostadienol (130%) and total sterol (58.7%) in OE‐BjHMGS1 fruits over the vector control (Tables 1 and 2). Also, squalene (210%), cycloartenol (200%), 24‐methylene‐cycloartanol (15.6%), cycloeucalenol (99.5%), 24‐methylene‐lophenol (99.9%), campesterol (95.0%), β‐sitosterol (99.8%), stigmasterol (61.0%), Δ^7,22^‐ergostadienol (140%) and total sterol content (93.7%) increased in OE‐S359A fruits over the vector control (Tables 1 and 2). Accordingly, the average amounts of squalene (130%), cycloartenol (25.5%), cycloeucalenol (31.2%), campesterol (58.7%), β‐sitosterol (42.1%) and total sterol (22.1%) in OE‐S359A fruits were significantly higher than in OE‐BjHMGS1 (Tables 1 and 2).

**Table 1:** Sterol‐related intermediate, sterol, α‐tocopherol and carotenoid profiles of tomato HMGS‐OE fruits [μg/g dry weight (DW) for sterols and α‐tocopherol, mg/g DW for carotenoids]

Sterols	pSa13	430	445	622	625
Squalene	25.2 ± 0.55	24.5 ± 1.49	**45.7 ± 0.71** a	**58.9 ± 1.98** a ^**,**^ b	**99.1 ± 2.04** a ^**,**^ b
Cycloartenol	33.8 ± 2.79	**94.9 ± 5.22** a	**68.2 ± 1.40** a	**76.3 ± 0.60** a	**128.4 ± 8.39** a ^**,**^ b
24‐Methylene‐cycloartanol	83.2 ± 6.84	74.5 ± 1.16	**159.8 ± 4.84** a	**103.4 ± 0.05** a	89.0 ± 1.49
Cycloeucalenol	38.1 ± 1.42	**61.3 ± 3.08** a	**54.5 ± 1.65** a	**68.9 ± 1.00** a	**83.1 ± 5.68** a ^**,**^ b
24‐Methylene‐lophenol	52.2 ± 1.69	**93.0 ± 4.09** a	**112.7 ± 2.18** a	**86.7 ± 0.75** a	**122.1 ± 6.37**
Campesterol	78.2 ± 2.8	**98.0 ± 2.3** a	**94.2 ± 2.3** a	**152.8 ± 7.7** a ^**,**^ b	**152.2 ± 1.5** a ^**,**^ b
β‐Sitosterol	86.2 ± 2.5	**119.2 ± 1.7** a	**123.2 ± 3.5** a	**161.3 ± 4.3** a ^**,**^ b	**183.3 ± 4.8** a ^**,**^ b
Stigmasterol	105.2 ± 4.5	**169.9 ± 1.5** a	**140.9 ± 2.5** a	**155.4 ± 1.9** a	**183.5 ± 2.6** a ^**,**^ b
Δ^7,22^‐Ergostadienol	43.8 ± 1.6	**109.2 ± 1.2** a	**88.5 ± 2.3** a	**86.7 ± 1.6** a	**123.5 ± 2.1** a ^**,**^ b
Total sterol	546.0 ± 12.3	**844.5 ± 10.9** a	**887.6 ± 10.7** a	**950.4 ± 10.0** a ^**,**^ b	**1164.2 ± 17.5** a ^**,**^ b
α‐Tocopherol	27.5 ± 3.2	**176.4 ± 4.4** a	**164.7 ± 6.2** a	**138.6 ± 13.4** a	**187.5 ± 3.6** a
Lycopene	3.0 ± 0.1	**10.7 ± 1.2** a	**9.3 ± 0.5** a	**6.7 ± 0.7** a ^**,**^ b	**6.0 ± 0.2** a ^**,**^ b
β‐Carotene	1.5 ± 0.06	**6.7 ± 0.09** a	**3.8 ± 0.1** a	**3.5 ± 0.3** a	**4.5 ± 0.09** a
Total carotenoids	4.5 ± 0.16	**17.4 ± 1.29** a	**13.1 ± 0.6** a	**10.2 ± 1.0** a ^**,**^ b	**10.5 ± 0.29** a ^**,**^ b

Two independent lines for each OE genotype were analysed. For OE‐wtBjHMGS1, lines 430 and 445 were tested. For OE‐S359A, lines 622 and 625 were tested. ^a^Indicates significant difference (*P* < 0.01 by the Student's *t*‐test) between HMGS‐OE and the vector (pSa13)‐transformed control; ^b^Indicates significant difference (*P* < 0.01 by the Student's *t*‐test) between OE‐wtBjHMGS1 and OE‐S359A. Total sterol content was calculated from the contents of all the components in the above table. Values are mean ± SD, *n* = 8 for sterol and 6 for carotenoid determination.

**Table 2:** Comparison in sterol‐related intermediate, sterol, α‐tocopherol and carotenoid composition in tomato fruits

	Elevation (%) in tomato fruits				
Sterol‐related intermediates and sterols	430 vs pSa13	445 vs pSa13	622 vs pSa13	625 vs pSa13	OE‐S359A vs OE‐wtBjHMGS1
Squalene	−2.9	**81.2**	**133.6**	**293.0**	**125.1**
Cycloartenol	**181.1**	**101.9**	**125.9**	**280.2**	6.5
24‐Methylene‐cycloartanol	−10.5	**92.1**	**24.3**	6.9	−17.9
Cycloeucalenol	**60.9**	**43.0**	**80.8**	**118.1**	**31.2**
24‐Methylene‐lophenol	**77.9**	**115.7**	**65.9**	**133.9**	1.6
Campesterol	**24.0**	**19.2**	**94.1**	**93.3**	**58.7**
β‐Sitosterol	**38.3**	**42.9**	**87.1**	**112.5**	**42.1**
Stigmasterol	**61.5**	**33.9**	**47.7**	**74.4**	9.0
Δ^7,22^‐Ergostadienol	**149.1**	**101.9**	**97.9**	**181.8**	6.4
Total sterol	**54.7**	**62.6**	**74.1**	**113.2**	**22.1**
α‐Tocopherol	**542.6**	**500.1**	**405.0**	**583.1**	−4.4
Lycopene	**256.6**	**211.0**	**123.7**	**98.4**	−**52.5**
β‐Carotene	**343.3**	**150.4**	**135.9**	**202.6**	−**31.4**
Total carotenoids	**277.1**	**184.5**	**122.8**	**128.1**	−**45.6**

Two independent lines for each OE genotype were analysed. For tomato OE‐wtBjHMGS1, lines 430 and 445 were tested. For tomato OE‐S359A, lines 622 and 625 were tested. The data presented for OE‐S359A in comparison with OE‐wtBjHMGS1 were calculated from an average of two lines (average of 622 and 625 for OE‐S359A in comparison with average of 430 and 445 for OE‐wtBjHMGS1). Bold font indicates significant (*P* < 0.05) % increases, in OE‐S359A (over OE‐wtBjHMGS1) and in OE‐wtBjHMGS1 and OE‐S359A (over the vector‐transformed control pSa13).

When the molecular mechanism for the accumulation of MVA‐derived compounds in HMGS‐OE fruits was investigated, the expression of *SlFPS1*,* SlGPS*,* SlSQS*,* SlSQE*,* SlCAS1*,* SlCYP85A1* and *SlCYP85A3* was found to be significantly higher in OE‐wtBjHMGS1 and OE‐S359A than in the control, with the exception of *SlSQS* in OE‐wtBjHMGS1 line 430 and *SlCYP85A1* in OE‐S359A line 625 (Figure S7a and b). *SlFPS1*,* SlGPS*,* SlSQS* and *SlCYP710A11* expression in OE‐S359A was much higher than in OE‐BjHMGS1 (Figure S7a and b). Conversely, the expression of *SlHMGR1*,* SlHMGR2* and *SlIPI* was down‐regulated in HMGS‐OE fruits (Figure S7a).

**Figure 6:**
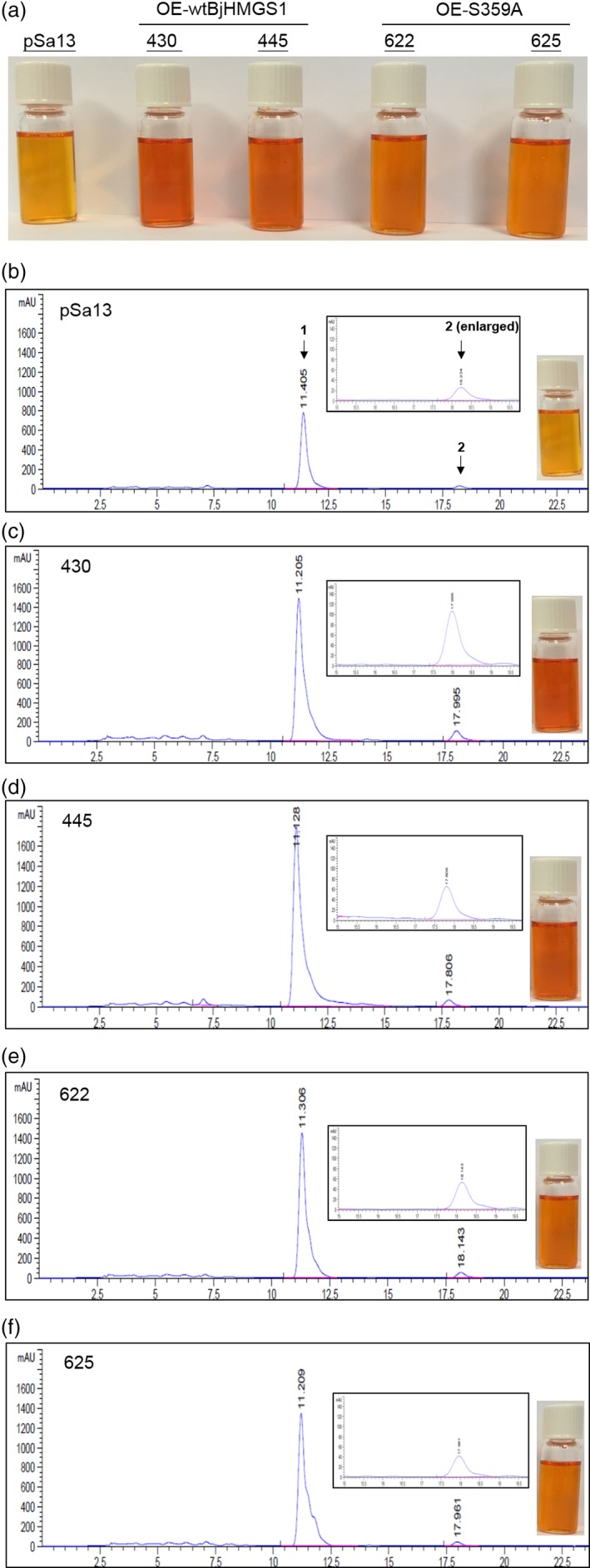
HPLC analysis of carotenoid content [μg/g dry weight (DW)] in 57 DAP mature tomato HMGS‐OE fruits. Carotenoids were extracted from the vector‐transformed control (pSa13), two independent lines of OE‐wtBjHMGS1 (430 and 445) and two independent lines of OE‐S359A (622 and 625). (a) Photographs of carotenoid extracts dissolved in chloroform; (b–f) HPLC chromatograms of carotenoids from pSa13, OE‐wtBjHMGS1 (430 and 450) and OE‐S359A (622 and 625), respectively. Peak 1, lycopene; peak 2, β‐carotene. The enlarged HPLC chromatogram of peak 2 and photograph of carotenoid extract dissolved in chloroform from each sample is presented in b–f.

More interestingly, a significant increase in vitamin E (α‐tocopherol) in tomato HMGS‐OE fruit lipid extracts was noted (Figure 5) from GC‐MS analysis. Vitamin E content in OE‐BjHMGS1 and OE‐S359A was significantly higher than in the vector control (Figure 5). Its content in HMGS‐OEs increased to 138.6–187.5 μg/g dry weight, in comparison with 27.5 μg/g dry weight in the control (Figure 5), representing average increases of 521% in OE‐BjHMGS1 and 494% in OE‐S359A (Figure 5). In HPLC analysis, a significant enhancement of carotenoids (lycopene and β‐carotene) in tomato HMGS‐OE fruit was observed (Figure 6). Lycopene and β‐carotene contents in OE‐BjHMGS1 and OE‐S359A were higher than in the control (Figure 6, Tables 1 and 2). Lycopene content in HMGS‐OEs increased to 6.0–10.7 mg/g dry weight, in comparison with 3.0 mg/g dry weight in the control (Table 1), representing average elevations of 234% in OE‐BjHMGS1 and 111% in OE‐S359A (Table 2). β‐Carotene content in HMGS‐OEs went up to 3.5–6.6 mg/g dry weight, in comparison with 1.5 mg/g dry weight in the control (Table 1), representing average increases of 247% in OE‐BjHMGS1 and 169% in OE‐S359A (Table 2). However, lycopene and total carotenoid contents in OE‐S359A were significantly lower than in OE‐BjHMGS1 (Figure 6, Tables 1 and 2). Lycopene and total carotenoid contents decreased by 53% and 46%, respectively, in OE‐S359A than in OE‐wtBjHMGS1 (Table 2). The elevation in vitamin E and carotenoids in OE‐BjHMGS1 and OE‐S359A fruits (Figure 5) coincided with an up‐regulation of plastidial *SlGPS* and *SlGGPPS1*, but not of *SlGGPPS2* in OE‐wtBjHMGS1 and OE‐S359A fruits (Figure S7c).

### Enhanced antioxidant activity of total carotenoids in tomato HMGS‐OE fruits

**Figure 7:**
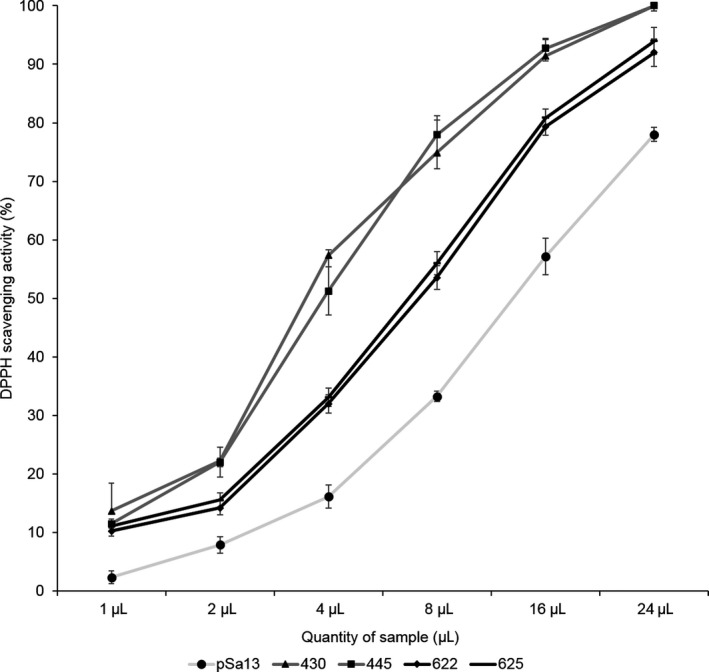
DPPH scavenging activities of carotenoids in 57 DAP mature tomato HMGS‐OE fruits. The vector‐transformed control (pSa13), two independent lines of OE‐wtBjHMGS1 (430 and 445) and two independent lines of OE‐S359A (622 and 625) were analysed. Values are means ± SD (*n* = 3).

As α‐tocopherol and carotenoids (lycopene and β‐carotene) (Azzi, 2007; DellaPenna, 2005; Fiedor and Burda, 2014; Shintani and DellaPenna, 1998) accumulated in tomato HMGS‐OE fruits (Tables 1 and 2), they were tested for antioxidant activity in comparison with the vector control. Tomato HMGS‐OE fruits displayed significantly (*P *<* *0.05) higher DPPH (1,1‐diphenyl‐2‐picrylhydrazyl) radical scavenging activities than the control (Figure 7). OE‐wtBjHMGS1 showed 257.9%–299.9% higher antioxidant activity than the control, while OE‐S359A was 89.5%–96.5% higher. OE‐wtBjHMGS1 possessed 82.1%–111.2% higher antioxidant activity than OE‐S359A (Figure 7), and this corresponded to a higher carotenoid content in OE‐wtBjHMGS1 than in OE‐S359A (Tables 1 and 2).

## Discussion

### Elevation of health‐promoting components achieved via metabolic engineering of BjHMGS1 in tomato fruit

Resembling tobacco HMGS‐OEs, tomato HMGS‐OEs showed an enhanced expression of *SlHMGR1*,* SlSQS*,* SlGGPPS1* and *SlCYP85A1* and an increase in growth accompanied by a decline in *SlHMGS2* and *SlIPI* expression. Furthermore, sterol content was elevated in HMGS‐OE tomato fruits, confirming that elevation of health‐promoting components was achieved via metabolic engineering of BjHMGS1 in tomato fruit and that extended phytosterol enhancement in fruits was attained using mutant S359A. New observations on tomato HMGS‐OE seedlings and fruits not previously reported for tobacco HMGS‐OE seedlings and leaves included:

- Up‐regulation of genes associated with the biosyntheses of C10, C15 and C20 universal precursors of isoprenoids, phytosterols and dolichols in seedlings;
- Up‐regulation of MEP‐, carotenoid‐ and vitamin E‐related genes, but down‐regulation of carotenoid‐related gene *SlPDS* and no apparent effect on monoterpene‐related gene (*SlMTS1*) expression in seedlings;
- Greater expression of MVA‐related genes, genes leading to C10, C15, C20 universal precursors of isoprenoids and sesquiterpene‐related genes in OE‐S359A than that of OE‐wtBjHMGS1 seedlings, corresponding to significant growth enhancement in 9‐week‐old OE‐S359A plants, suggesting that S359A overexpression affected the expression of these isoprenoid precursor‐ and sesquiterpene‐related genes;
- Up‐regulation of *SlFPS1*,* SlSQE*,* SlCAS1*,* SlCYP85A1*,* SlCYP85A3*,* SlGPS* and *SlGGPPS1* in HMGS‐OE fruits, resulting in higher amounts of health‐promoting components including squalene, vitamin E (α‐tocopherol) and carotenoids; and
- Elevation of fruit squalene and phytosterols in OE‐S359A in comparison with OE‐wtBjHMGS1, which was attributed to higher expression of *SlHMGR2*,* SlFPS1*,* SlGPS*,* SlSQS* and *SlCYP710A11* in OE‐S359A fruits.

### HMGS regulates isoprenoid biosynthesis genes in tomato HMGS‐OE seedlings

It has been reported that *NtHMGR1* is a housekeeping gene, while *NtHMGR2* is stress‐inducible (Hemmerlin *et al*., 2004; Merret *et al*., 2007). This corresponds well to the up‐regulation of *NtHMGR1*, but not *NtHMGR2*, in tobacco HMGS‐OE seedlings (Liao *et al*., 2014b). In tomato, *SlHMGR1* is known to be highly expressed at the early stage of fruit development, while *SlHMGR2* is confined to fruit maturation and ripening (Narita and Gruissem, 1989). Not surprisingly, *SlHMGR1*, but not *SlHMGR2*, expression closely coincided with growth‐dependent phytosterol biosynthesis (Rodríguez‐Concepción and Gruissem, 1999). In this study, the differential expression of *SlHMGR1* and *SlHMGR2* in tomato seedlings (Figure 3) resembles that of tomato fruits (Narita and Gruissem, 1989; Rodríguez‐Concepción and Gruissem, 1999).

Besides *SlHMGR2*,* SlIPI* was significantly down‐regulated in tomato HMGS‐OE seedlings (Figure 3), like *NtIPI1* in transgenic tobacco HMGS‐OEs (Liao *et al*., 2014b). Besides IPI, plastid‐localized SlCPT1 and SlCPT2 prefer DMAPP as a substrate (Akhtar *et al*., 2013), and their mRNA expression in tomato OE‐S359A seedlings was significantly higher than in OE‐wtBjHMGS1 (Figure 3), corresponding to better growth (Figure 2). Interestingly, the increase in *SlCPT1* expression was greater than that of *SlCPT2* in these tomato HMGS‐OE seedlings (Figure S5a), implying that HMGS overexpression exerted a stronger effect on *SlCPT1*.

*SlFPS1* has been proposed to play an important role in early fruit development as well as cell division and elongation (Gaffe *et al*., 2000). Simultaneous silencing of both FPS in Arabidopsis reduced sterol content and retarded seedling growth (Manzano *et al*., 2016). While *NtFPPS* expression was slightly elevated in tobacco seedlings (Liao *et al*., 2014b), *SlFPS1* expression in tomato OE‐wtBjHMGS1 seedlings remained unchanged and was up‐regulated in OE‐S359A (Figure 3). Higher *SlFPS1* expression observed in OE‐S359A in comparison with OE‐wtBjHMGS1 tomato seedlings (Figure 3) coincided well with a positive growth effect in 9‐week‐old OE‐S359A, suggesting that S359A overexpression was more effective in enhancing *SlFPS1* expression.

Squalene, product of SQS (Abe *et al*., 1993; Devarenne *et al*., 1998, 2002; Seo *et al*., 2005), has been reported to confer health benefits because it possesses antitumour properties (Mathews, 1992; Newmark, 1997; Smith, 2000), and abilities in quenching singlet oxygen (Kohno *et al*., 1995) and reducing phenobarbital, theophylline and strychnine in animals (Kamimura *et al*., 1992). The overexpression of *Panax ginseng* SQS1 enhanced sterol and ginsenoside contents in transgenic *P. ginseng* roots (Lee *et al*., 2004). Transgenic Arabidopsis overexpressing *Glycine max* SQS1 demonstrated significant elevation in seed sterols (Nguyen *et al*., 2013). The silencing of *Withania somnifera SQS* down‐regulated downstream sterol pathway genes, reduced squalene, and sterol content and caused a dwarf phenotype (Singh *et al*., 2015). In this study, an enhanced effect of S359A on squalene production (Figure 5, Tables 1 and 2) mirrored the benefits seen in *P. ginseng* and Arabidopsis (Lee *et al*., 2004; Nguyen *et al*., 2013). The increase in *SlSQS* expression correlated with enhanced squalene and sterol accumulation in tomato HMGS‐OE fruits and higher *SlSQS* expression in OE‐S359A tomato seedlings and fruits corresponded well with elevated squalene and sterol contents, consistent with greater expression of *NtSQS and AtSQS* (Liao *et al*., 2014b; Wang *et al*., 2012). However, reports of a dwarf phenotype from the overexpression of a truncated yeast SQS or codon‐optimized Flag‐tagged yeast SQS in tobacco (Pasoreck *et al*., 2016; Wu *et al*., 2012) suggest differences do occur between applications of plant and yeast SQS.

*SlGGPPS1* was highly expressed in tomato leaves in contrast to *SlGGPPS2*, which was induced in fruits and flowers (Ament *et al*., 2006). Both *SlGGPPS1* and *SlGGPPS2* expression was up‐regulated in tomato HMGS‐OE seedlings (Figure S5), but only *NtGGPPS2* was elevated in several lines of tobacco HMGS‐OE seedlings, while *NtGGPPS1*,* NtGGPPS3* and *NtGGPPS4* remained down‐regulated in all previous lines tested (Liao *et al*., 2014b). These results suggest that HMGS overexpression produced inconsistent effects on *GGPPS* expression in tomato and tobacco seedlings. It appears that HMGS overexpression caused a stronger positive effect on *GGPPS* expression in tomato seedlings in comparison with tobacco. Ruiz‐Sola *et al*. (2016) identified one *GGPPS* gene encoding two differentially targeted (plastidial and cytosol) GGPPSs in Arabidopsis, implying that such could also occur in tomato and tobacco.

### Co‐up‐regulation of BR‐, CK‐ and dolichol‐related genes in tomato HMGS‐OEs

In higher plants, besides sterols, BRs and CKs are essential in growth and development (He *et al*., 2003; Howell *et al*., 2003; Li *et al*., 1996; Shani *et al*., 2010; Vriet *et al*., 2012; Wang *et al*., 2012), while dolichol is important in protein glycosylation (Zhang *et al*., 2008). BR‐related genes were up‐regulated in Arabidopsis and tobacco HMGS‐OEs (Liao *et al*., 2014b; Wang *et al*., 2012). A BR (*SlCYP85A3*)‐ and a dolichol (*SlCPT3*)‐related gene were dramatically up‐regulated in tomato HMGS‐OE seedlings and more highly expressed in OE‐S359A than in OE‐wtBjHMGS1 (Figure S4a and c), indicating an effect from HMGS overexpression. In *Caenorhabditis elegans*,* HMGS* is significant in the miRNA pathway by regulating the function of many miRNAs during development (Shi and Ruvkun, 2012). MVA‐derived dolichols, which are involved in *N*‐glycosylation, are essential for the activity of miRNAs in silencing their target mRNAs (Shi and Ruvkun, 2012). More experiments are needed to address the relationship between HMGS and dolichol accumulation in plants.

### The overexpression of cytosolic HMGS promotes carotenoid and vitamin E formation in plastids

The overexpression of Arabidopsis HMGR in *Lavandula latifolia* and that of cytosolic isopentenyl phosphate kinase in Arabidopsis and tobacco increased both MVA‐derived sterols and MEP‐derived monoterpenes and sesquiterpenes (Henry *et al*., 2015; Muñoz‐Bertomeu *et al*., 2007). *Salvia miltiorrhiza* HMGR overexpression in hairy roots enhanced MEP‐associated diterpene tanshinone accumulation (Kai *et al*., 2011). In this study, MEP‐related genes (*SlDXS1*,* SlDXS2* and *SlDXR*), most carotenoid‐related genes (*SlPSY‐1*,* SlPSY‐2*,* SlZDS*,* SlCRTISO*,* SlLCY‐B*,* SlCYC‐B* and *SlLCY‐E* with the exception of *SlPDS*) and vitamin E‐related genes (*SlGGPPR* and *SlGMTT*) were up‐regulated in tomato HMGS‐OE seedlings (Figures 4 and S6), suggesting that HMGS overexpression in the cytosol can affect the biosynthesis of plastidial MEP‐related isoprenoids including carotenoids and vitamin E. Indeed, total carotenoids increased in tomato HMGS‐OE fruits (Figure 6, Tables 1 and 2) and lycopene content and total carotenoids were much higher in OE‐wtBjHMGS1 than in OE‐S359A (Figure 6, Tables 1 and 2). In contrast, the expression of *SlCRTISO* and *SlCYC‐B* in tomato OE‐S359A seedlings was significantly higher than in OE‐wtBjHMGS1 (Figure S6). These results implied that differential carotenoid‐related gene expression had probably occurred between tomato HMGS‐OE seedlings and fruits, or post‐transcriptional/post‐translational regulation may have taken effect. Furthermore, increased HMGS enzyme activity in OE‐S359A resulted in enhanced MVA‐derived squalene and phytosterols but not MEP‐derived carotenoids and vitamin E (Tables 1 and 2).

Vitamin E consists of two forms, tocopherols and tocotrienols (Brigelius‐Flohé and Traber, 1999). Previous studies manipulated enzymes in the vitamin E biosynthetic pathway to increase leaf (37%: 10‐fold) and seed (18%–1500%) vitamin E content in model plants Arabidopsis and tobacco, as well as crop plants including canola, soybean, corn, lettuce, potato and sunflower (Chen *et al*., 2006 and references cited therein; Del Moral *et al*., 2013; DellaPenna, 2005 and references cited therein; Vom Dorp *et al*., 2015). Also, the overexpression of zeaxanthin epoxidase in potato resulted in a two‐ to threefold elevation in α‐tocopherol (Römer *et al*., 2002). However, the metabolic engineering of the MVA pathway or HMGS for α‐tocopherol accumulation in a fruit crop had not been reported. We successfully demonstrated herein that manipulation of HMGS from the MVA pathway in tomato led to dramatic increase (~5‐fold) in fruit α‐tocopherol (Figure 5). This confirms that the overexpression of a cytosolic HMGS could cause an increase in plastidial GGPP‐derived vitamin E. There is evidence of cross‐talk between the MVA and MEP pathways in up‐regulated *SlGGPPS1* expression in HMGS‐OE tomato fruits, promoted α‐tocopherol production. It is worth noting that there was no significant difference in the level of α‐tocopherol between OE‐wtBjHMGS1 and OE‐S359A, indicating that S359A was not superior to wtBjHMGS1 in enhancing α‐tocopherol production. Hence, metabolic engineering of HMGS from the MVA pathway can provide an alternative strategy in elevating α‐tocopherol production in a fruit crop. Also, the tomato HMGS‐OE fruits generated in this study present potential as an emerging form of beneficial food enriched in health‐promoting components including squalene, phytosterols, carotenoids and α‐tocopherol.

## Experimental procedures

### Plant materials and growth conditions

Wild‐type tomato (*Lycopersicon esculentum* Mill. cv. UC82B) seeds were obtained from Dr. WK Yip, The University of Hong Kong. Tomato seeds were surface‐sterilized in 75% ethanol for 1 min, rinsed thrice in sterilized water, soaked in 25% Clorox for 10 min and rinsed four times with sterilized water. Seeds were transferred to MS medium for 2 days at 4 °C before being moved to a tissue culture room for germination and seedling development. Tomato plants were grown at 25 °C (16‐h light)/22 °C (8‐h dark).

### Generation and characterization of transgenic tomato overexpressing HMGS

Plasmids pBj134 (wt‐BjHMGS1) and pBj136 (S359A) from Wang *et al*. (2012) were used for *Agrobacterium*‐mediated tomato transformation (Mathews *et al*., 2003) with vector control pSa13 (Xiao *et al*., 2008). T_1_ transgenic tomato seeds were screened on MS with 50 μg/ml kanamycin and analysed by PCR followed by DNA sequencing (Liao *et al*., 2014b; Wang *et al*., 2012). T_2_ homozygous lines with single copy of BjHMGS1/S359A were analysed in mRNA and protein expression, plant growth and metabolite composition. Tomato total protein was extracted (Chye *et al*., 1999) from 3‐week‐old fresh tomato leaves and protein concentration measured (Bradford, 1976). Western blot analysis was conducted as described previously (Liao *et al*., 2014b; Wang *et al*., 2012; Xiao *et al*., 2010). Antibodies against BjHMGS1 were used in Western blot analysis (Wang *et al*., 2012). *Eco*RI‐digested tomato genomic DNA (40 μg) from 4‐week‐old leaves was separated on agarose gel (0.7%) by electrophoresis. Southern blot analysis (Southern, 2006) was performed using a digoxigenin‐labelled full length of *BjHMGS1* cDNA probe generated by primer pair ML264 and ML276 (Wang *et al*., 2012). Primers are listed in Table S1.

### Semiquantitative reverse transcription PCR (RT‐PCR)

Total RNA from 3‐week‐old tomato seedlings was extracted using RNeasy Plant Mini Kit (Qiagen, Hilden, Germany) followed by DNase I treatment (Qiagen). First‐strand cDNA was synthesized from 5 μg total RNA from 3‐week‐old tomato seedlings using the SuperScript First‐Strand Synthesis System (Invitrogen, Carlsbad, CA, USA). Semiquantitative reverse transcription PCR (RT‐PCR) was conducted using the PCR System (Bio‐Rad, Hercules, USA) with *BjHMGS1*‐specific primer pair (ML1666 and ML1667) and tomato *ACTIN* primer pair (ML1688 and ML1689). Tomato *ACTIN* (*SlACTIN*), which has been previously tested (EI‐Sharkawy *et al*., 2016; Melilli *et al*., 2014), was used as an internal control to estimate the amount of RNA in each sample. The conditions for PCR were as follows: denaturation at 95 °C for 5 min, followed by 35 cycles of amplification (95 °C for 15 s, 60 °C for 20 s and 72 °C for 20 s) and extension at 72 °C for 10 min. The experiment was repeated two times. Primers for RT‐PCR are listed in Table S1.

### Quantitative reverse transcription PCR

Total RNA from 3‐week‐old tomato seedlings or mature red tomato fruits aged 57 days after pollination (DAP; breaker+15) was extracted using RNeasy Plant Mini Kit (Qiagen). The RNA (5 μg) was treated using DNase I (Qiagen) before reverse‐transcribed into first‐strand cDNA using the SuperScript First‐Strand Synthesis System (Invitrogen). Quantitative reverse transcription PCR (qRT‐PCR) was carried out with a StepOne Plus Real‐time PCR System (Applied Biosystems, Foster City, CA, USA) and FastStart Universal SYBR Green Master (Roche, Mannheim, Germany). The conditions for qRT‐PCR were as follows: denaturation at 95 °C for 10 min, followed by 40 cycles of 95 °C for 15 s and 60 °C for 1 min. Three experimental replicates for each reaction were carried out using gene‐specific primers, and tomato *ACTIN* was used as the internal control. The comparative C_T_ method was used to analyse the qRT‐PCR data (Schmittgen and Livak, 2008). The relative expression was normalized to *SlACTIN*, and the relative mRNA level in each HMGS‐OE line in comparison with the empty vector control from three independent experiments was presented on the *y*‐axis. Significant differences in the relative mRNA levels between different samples were analysed by the Student's *t*‐test. Primers for qRT‐PCR are listed in Table S1.

### Measurements of growth rate

Phenotypic changes in tomato plants were analysed (Johnston and Dore, 1929). Single‐copy T_2_ homozygous plants were compared in plant growth. Four‐day‐old tomato seedlings were moved to fresh MS plates and grown for 8 days. Twelve‐day‐old tomato seedlings of similar size were transferred to soil for further analysis; height measurements of 5‐week‐old and 9‐week‐old tomato plants were taken. For each OE construct, two independent lines were tested. Thirty plants per individual line were used for measurements in height.

### Extraction and GC‐MS/HPLC/antioxidant activity analysis of sterols, intermediates, vitamin E and carotenoids

Extraction and analysis of sterols, intermediates, vitamin E (Babiychuk *et al*., 2008; Liao *et al*., 2014b; Schaller *et al*., 1995; Wang *et al*., 2012) and carotenoids (Fraser *et al*., 2000; Zanfini *et al*., 2010) in transgenic tomato fruits were performed. Antioxidant activity analysis was conducted by DPPH (1,1‐diphenyl‐2‐picrylhydrazyl) (Blois, 1958). See details for these analyses in Supplementary Methods.

### Statistical analysis

Significant differences in data between different samples were analysed by the Student's *t*‐test.

## Conflict of interest

The authors declare no conflict of interest.

## Supporting information


**Figure S1** PCR analysis on representative transgenic tomato HMGS‐OEs.
**Figure S2** Southern blot analysis of representative transgenic tomato HMGS‐OEs.
**Figure S3** QRT‐PCR analysis on the expression of sesquiterpene‐related genes in tomato HMGS‐OE seedlings.
**Figure S4** QRT‐PCR analysis on the expression of BR‐, cytokinin‐ and dolichol‐related genes in tomato HMGS‐OE seedlings.
**Figure S5** QRT‐PCR analysis on the expression of C10 and C20 universal precursors of isoprenoid‐, and monoterpene‐related genes in tomato HMGS‐OE seedlings.
**Figure S6** QRT‐PCR analysis on the expression of MEP‐derived carotenoid‐ and vitamin E‐related genes in tomato HMGS‐OE seedlings.
**Figure S7** QRT‐PCR analysis on the expression of genes downstream of *HMGS* and plastidial *GGPPSs* in tomato HMGS‐OE fruits.
**Table S1** Oligonucleotide primers used in this study.
**Data S1** Supplementary Methods.
**Data S2** Supplementary Result.
