# Supplementary material for: Improved fruit α‐tocopherol, carotenoid, squalene and phytosterol contents through manipulation of Brassica juncea 3‐HYDROXY‐3‐METHYLGLUTARYL‐COA SYNTHASE1 in transgenic tomato
Source: Plant Biotechnol J. 2017 Oct 17;16(3):784–96. doi: 10.1111/pbi.12828 (PMC5814594; doi:10.1111/pbi.12828)
Supplement: Supplementary file 1 — Figure S1 PCR analysis on representative transgenic tomato HMGS‐OEs. Figure S2 Southern blot analysis of representative transgenic tomato HMGS‐OEs. Figure S3 QRT‐PCR analysis on the expression of sesquiterpene‐related genes in tomato HMGS‐OE seedlings. Figure S4 QRT‐PCR analysis on the expression of BR‐, cytokinin‐ and dolichol‐related genes in tomato HMGS‐OE seedlings. Figure S5 QRT‐PCR analysis on the expression of C10 and C20 universal precursors of isoprenoid‐, and monoterpene‐related genes in tomato HMGS‐OE seedlings. Figure S6 QRT‐PCR analysis on the expression of MEP‐derived carotenoid‐ and vitamin E‐related genes in tomato HMGS‐OE seedlings. Figure S7 QRT‐PCR analysis on the expression of genes downstream of HMGS and plastidial GGPPSs in tomato HMGS‐OE fruits. Table S1 Oligonucleotide primers used in this study. Data S1 Supplementary Methods. Data S2 Supplementary Result. [file PBI-16-784-s001.docx]

**Supplementary Materials**

**SUPPLEMENTARY METHODS**

**Extraction and GC-MS analysis of sterols, intermediates and vitamin E**

For sterol profiling, 200 mg of freeze-dried materials from 57 d DAP mature red tomato fruits was used. Extraction and quantitative analysis of sterols were performed following previous methods (Babiychuk et al., 2008; Liao et al., 2014b; Schaller et al., 1995; Wang et al., 2012). For each OE genotype, two independent tomato lines were analysed. For each independent tomato HMGS-OE line, four independent samples were used to extract sterols. During GC-MS analysis, two injections were done for each sample. Lupenyl-3,28-diacetate was applied as the internal standard; its peak area in GC-MS was used to calculate the relative content of sitosterol, campesterol, stigmasterol, several related intermediates and vitamin E.

**Extraction and HPLC analysis of carotenoids**

Extraction of carotenoids was performed accordingly to Fraser et al. (2000). Methanol (200 µl) was added into a tube containing 20 mg of freeze-dried material from 57 d DAP mature red tomato fruits, mixed and incubated at 4^o^C for 5 min. Tris-HCl (200 µl) (50 mM, pH 7.5) was then added and incubated at 4^o^C for 10 min. Subsequently, chloroform (800 µl) was added and incubated on ice for 10 min followed by centrifugation at 3000 *g* at 4^o^C for 5 min. The hypophase was collected and transferred to a new glass tube. The extraction of carotenoids using chloroform was repeated once. The pooled chloroform extracts were dried under nitrogen gas and re-dissolved in 500 µl of chloroform/methanol (1:1, v/v). The solution was passed through a 0.22 µm filter before HPLC analysis following Zanfini et al. (2010) and Fraser et al. (2000) with some minor modifications, using an Agilent 1260 Infinity equipped with (Diode Array Detector) DAD/ (Fluorescence Detector) FLD. A ACE reverse-phase C18, 5 µm column (250 mm × 4.6 mm) with mobile phase consisting of acetonitrile, methanol, and dichloromethane (60:25:15) was used. Column temperature was held at 25^o^C and the flow rate was 1 ml/min. Chromatograms were recorded at 450 nm. Lycopene and β-carotene were identified and quantified using calibration curves of authentic lycopene (Sigma, L9879) and β-carotene (Sigma, C9750) standards.

**Antioxidant activity analysis**

The DPPH (1,1-diphenyl-2-picrylhydrazyl) radical scavenging activities of total carotenoid from tomato HMGS-OE fruits were determined according to the method of Blois (1958). Different volumes (1, 2, 4, 8, 16 and 24 µl) of tomato fruit carotenoid extract were diluted with methanol to 0.5 ml and mixed with an equal volume of DPPH (0.2 mM in methanol) solution. The mixture was incubated at room temperature for 30 min before absorbance was measured at 517 nm with a spectrophotometer (GE Healthcare GeneQuant 100). The percentage of radical scavenging activity of total carotenoid was calculated using this formula, DPPH radical scavenging activity (%) = [1-(absorbance of the solution with extracts/absorbance of the solution without extracts)]×100 (%). The IC_50_ value, which represents the amount of antioxidant activity required to reduce DPPH by 50%, was determined using dose-response curves. The DPPH radical scavenging activities of total carotenoids between HMGS-OE and the vector control were compared using the IC_50_ value from each sample.

**SUPPLEMENTARY RESULT**

**Characterisation of transgenic tomato overexpressing wt and mutant BjHMGS1**

Putative tomato HMGS-OEs, designated as OE-wtBjHMGS1 lines (401, 403, 405, 406, 421, 423, 430, 442 and 445) and OE-S359A lines (605, 607, 608, 612, 613, 614, 615, 622 and 625) were analysed by PCR (Figure S1) followed by DNA sequence analysis on the PCR product to confirm the presence of wt and mutant BjHMGS1 (S359A). Western blot analysis using anti-BjHMGS1 antibodies (Wang et al., 2012) confirmed the overexpression of HMGS by detection of a 52.4-kDa cross-reacting HMGS band on PCR-positive HMGS-OEs (Figure 2a-b). Following Southern blot analysis to identify single-insertional lines (Figure S2), two independent single-insertional lines of OE-wtBjHMGS1 (430 and 445) and OE-S359A (622 and 625) were selected for subsequent experiments. Semi-quantitative RT-PCR confirmed that these single-insertion lines of OE-wtBjHMGS1 (430 and 445) and OE-S359A (622 and 625) showed *BjHMGS1* mRNA expression, while the vector-transformed control (pSa13) did not (Figure 2c).


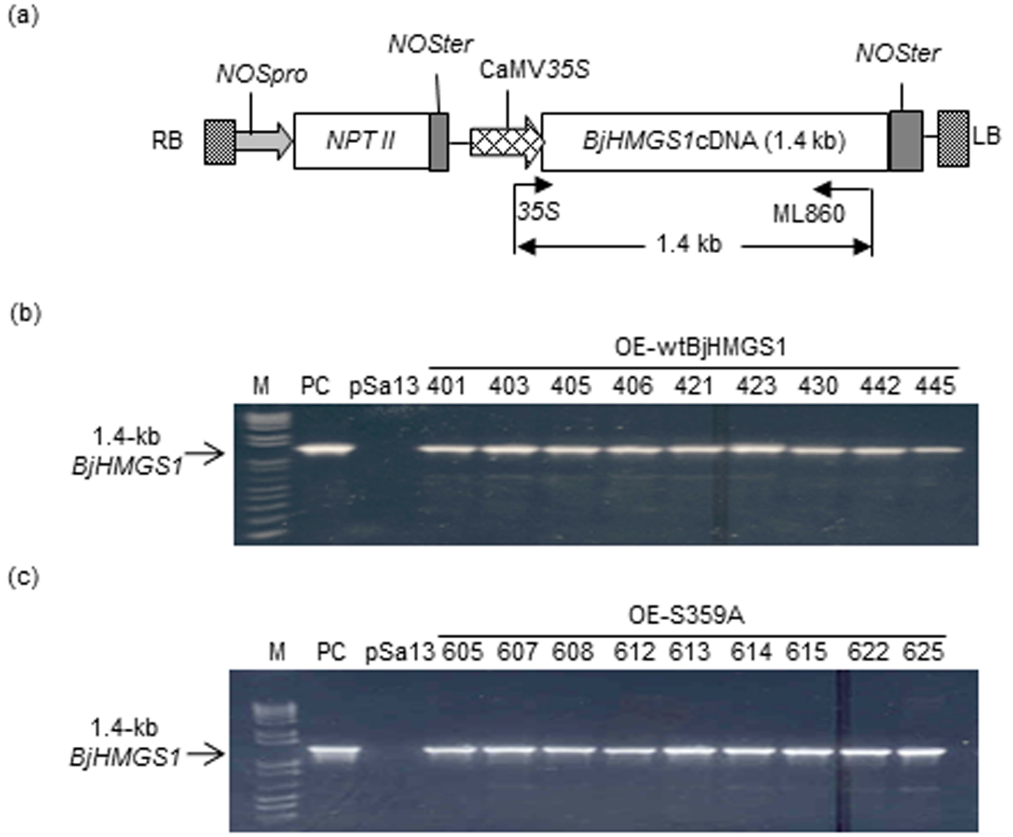


**Figure S1.** PCR analysis on representative transgenic tomato HMGS-OEs. (a) Schematic map of transformation vector pBj134 (wtBjHMGS1) and pBj136 (S359A) indicating primer location. *BjHMGS1* wild-type and mutant S359A were derived from plasmids pBj134 (wtBjHMGS1) and pBj136 (S359A), respectively (Wang *et al*., 2012). Plasmid pBj136 (S359A) contains a single nucleotide mutation resulting in amino acid substitution S to A at position 359 of BjHMGS1. CaMV*35S*: Cauliflower Mosaic Virus *35S* promoter; *NOSpro*: nopaline synthase (*NOS*) promoter**;** *NOSter*: *NOS* terminator; *NPTII*: gene encoding neomycin phosphotransferase II conferring resistance to kanamycin; RB: right border of T-DNA; LB: left border of T-DNA. *35S*: *35S* promoter 3’-end forward primer; ML860: *BjHMGS1-*specific reverse primer. Putative tomato HMGS-OEs were designated as OE-wtBjHMGS1 lines (401, 403, 405, 406, 421, 423, 430, 442 and 445) and OE-S359A lines (605, 607, 608, 612, 613, 614, 615, 622 and 625). (b) PCR analysis to detect the presence of wild-type *BjHMGS1* cDNA in transgenic tomato. Representative lines are shown here. M, 1 kb marker; PC, positive control (plasmid of pBj134); pSa13, negative control (plasmid of vector pSa13). (c) PCR analysis to detect the presence of mutant *BjHMGS1* (S359A) cDNA in transgenic tomato. Representative lines are shown here. M, 1 kb marker; PC, positive control (plasmid of pBj134); pSa13, negative control (plasmid of vector pSa13).


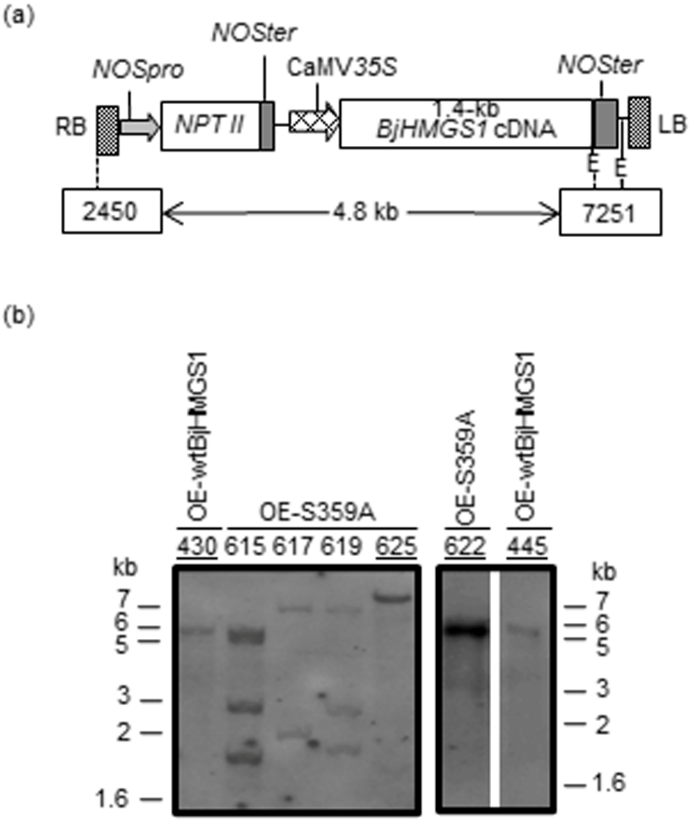


**Figure S2.** Southern blot analysis of representative transgenic tomato HMGS-OEs. (a) Schematic map of transformation vector pBj134 (wtBjHMGS1) and pBj136 (S359A) indicating *Eco*RI (E) sites. *BjHMGS1* wild-type and mutant S359A were derived from plasmids pBj134 (wt*BjHMGS1*) and pBj136 (S359A). Plasmid pBj136 (S359A) contains a single nucleotide mutation resulting in amino acid substitution S to A at position 359 of BjHMGS1. CaMV*35S***:** Cauliflower Mosaic Virus *35S* promoter; *NOSpro*: nopaline synthase (*NOS*) promoter**;** *NOSter*: *NOS* terminator; *NPTII*: gene encoding neomycin phosphotransferase II conferring resistance to kanamycin; RB: right border of T-DNA; LB: left border of T-DNA. (b) Southern blot analysis of tomato leaf DNA digested with *Eco*RI (E) and probed with digoxigenin-labeled *BjHMGS1* full-length cDNA in two representative blots. The hybridising band for a single insertional event is expected to be greater than 4.8 kb (see map in (a)). When representative tomato OE-wtBjHMGS1 (430 and 445) and OE-S359A lines (615, 617, 619, 622 and 625) were tested, two single-insertion lines of OE-wtBjHMGS1 (430 and 445 as underlined) and OE-S359A (622 and 625 as underlined) emerged and were used in further experiments. The black rectangles indicate lanes derived from the same gel. White lines have been inserted between lanes that have been spliced together from the same original gel/blot.


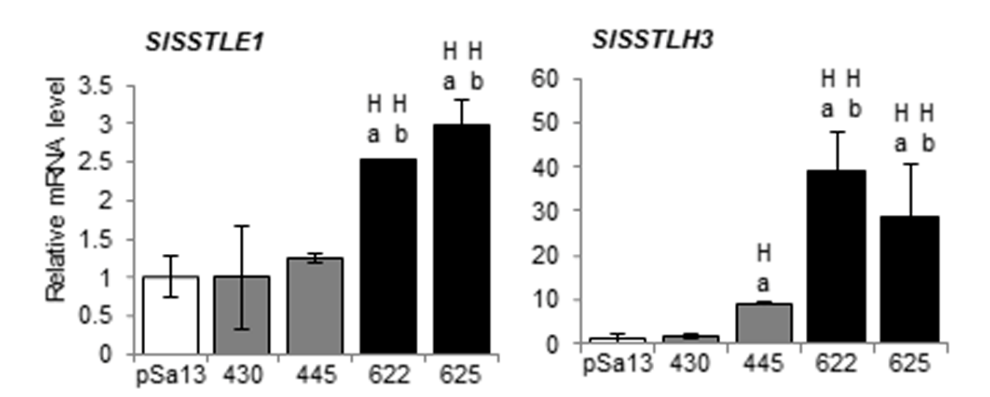


**Figure S3.** QRT-PCR analysis on the expression of sesquiterpene-related genes in tomato HMGS-OE seedlings. Total RNA was extracted from 3-week-old tomato seedlings of the vector (pSa13)-transformed control, OE-wtBjHMGS1 lines (430 and 445) and OE-S359A lines (622 and 625). H, value higher than the control; L, value lower than the control. Values are means ± SD (n=3). a indicates significant difference between HMGS-OE and the vector (pSa13)-transformed control; b indicates significant difference between OE-wtBjHMGS1 and OE-S359A (*P*< 0.05, Student’s *t*-test).


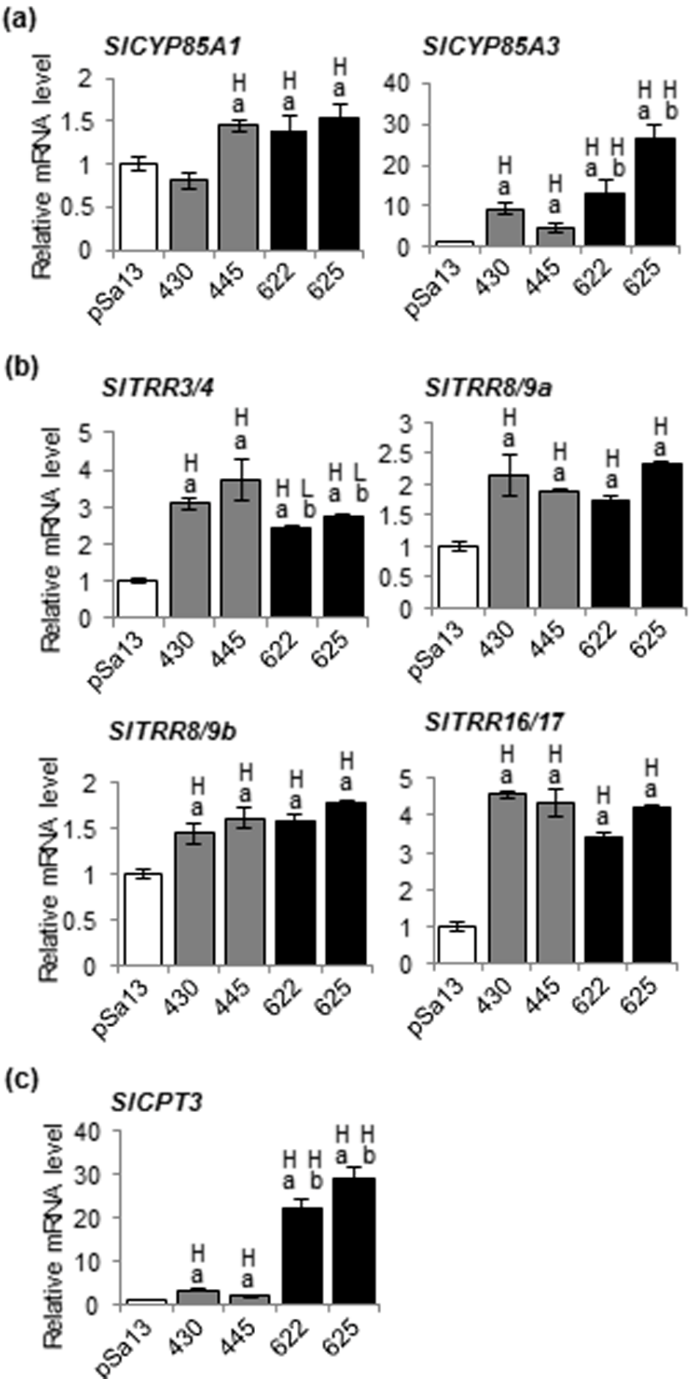


**Figure S4.** QRT-PCR analysis on the expression of BR-, cytokinin- and dolichol-related genes in tomato HMGS-OE seedlings. Total RNA was extracted from 3-week-old tomato seedlings of the vector (pSa13)-transformed control, OE-wtBjHMGS1 lines (430 and 445) and OE-S359A lines (622 and 625). H, value higher than the control; L, value lower than the control. Values are means ± SD (n=3). a indicates significant difference between HMGS-OE and the vector (pSa13)-transformed control; b indicates significant difference between OE-wtBjHMGS1 and OE-S359A (*P*< 0.05, Student’s *t*-test).


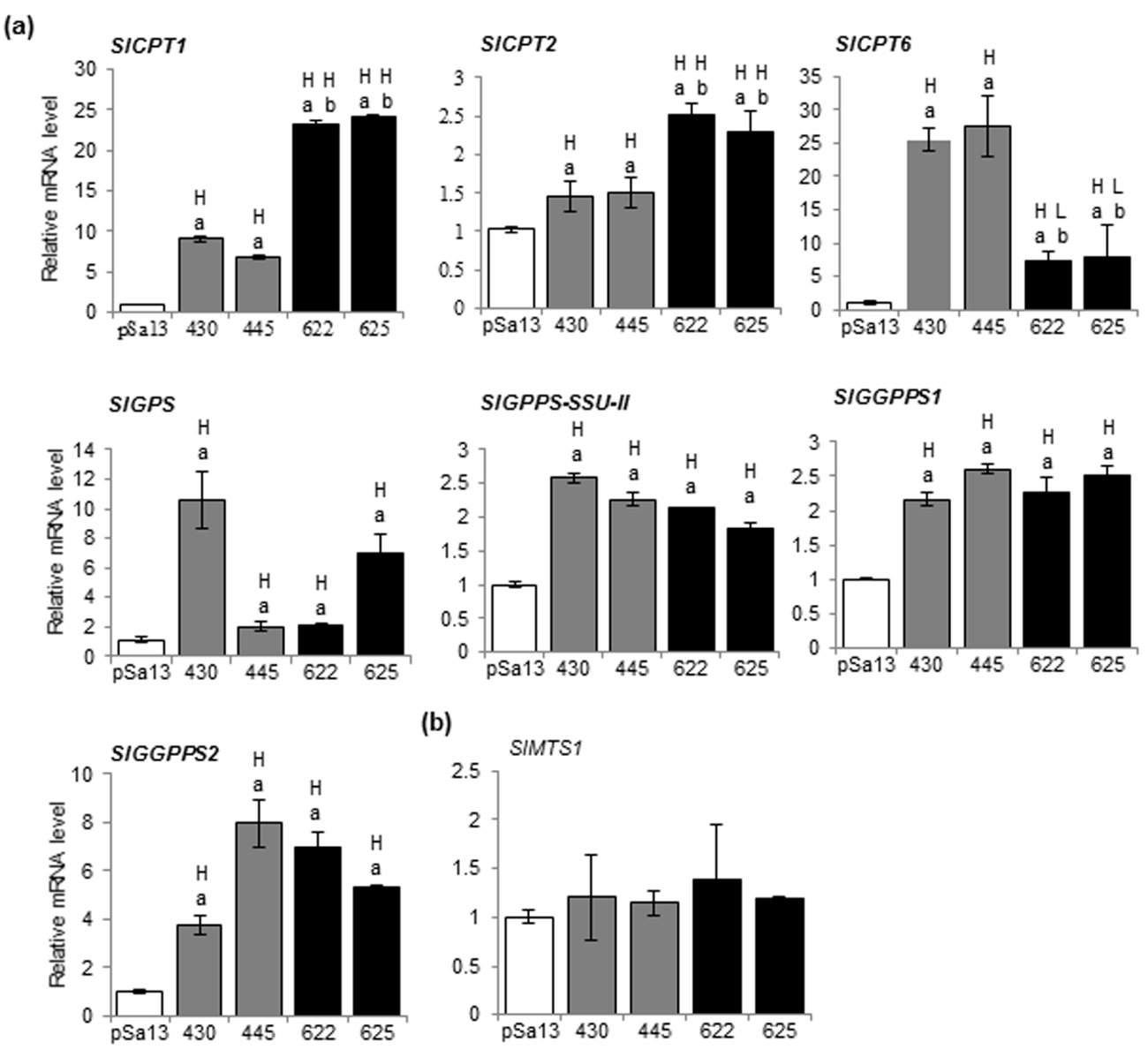


**Figure S5.** QRT-PCR analysis on the expression of C10, C15 and C20 universal precursors of isoprenoid-, and monoterpene-related genes in tomato HMGS-OE seedlings. Total RNA was extracted from 3-week-old tomato seedlings of the vector (pSa13)-transformed control, OE-wtBjHMGS1 lines (430 and 445) and OE-S359A lines (622 and 625). H, value higher than the control; L, value lower than the control. Values are means ± SD (n=3). a indicates significant difference between HMGS-OE and the vector (pSa13)-transformed control; b indicates significant difference between OE-wtBjHMGS1 and OE-S359A (*P*< 0.05, Student’s *t*-test).


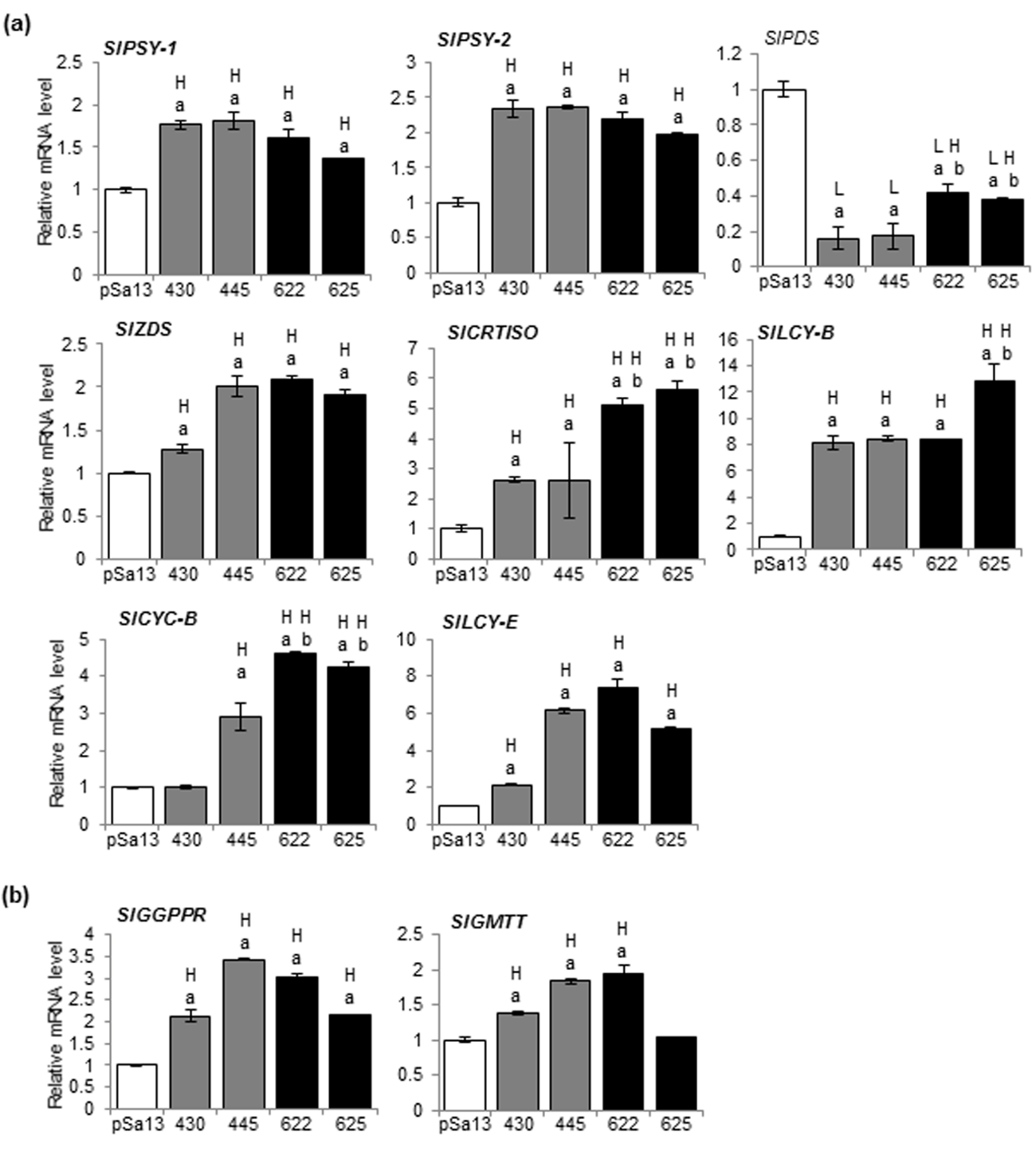


**Figure S6.** QRT-PCR analysis on the expression of MEP-derived carotenoid- and vitamin E-related genes in tomato HMGS-OE seedlings. Total RNA was extracted from 3-week-old tomato seedlings of the vector (pSa13)-transformed control, OE-wtBjHMGS1 lines (430 and 445) and OE-S359A lines (622 and 625). H, value higher than the control; L, value lower than the control. Values are means ± SD (n=3). a indicates significant difference between HMGS-OE and the vector (pSa13)-transformed control; b indicates significant difference between OE-wtBjHMGS1 and OE-S359A (*P*< 0.05, Student’s *t*-test).


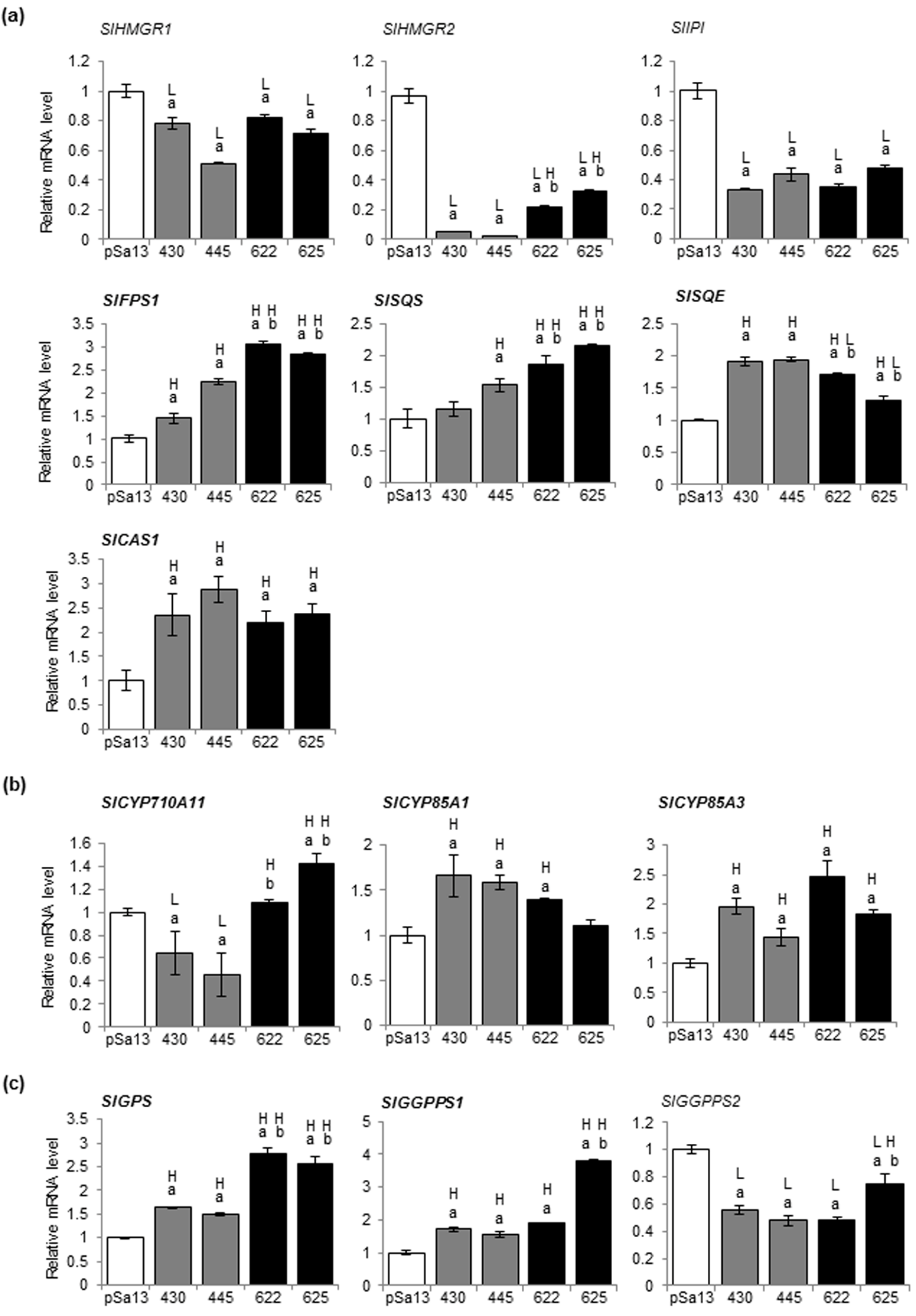


**Figure S7.** QRT-PCR analysis on the expression of genes downstream of *HMGS* and plastidial *GGPPSs* in tomato HMGS-OE fruits. Total RNA was extracted from 57 DAP mature tomato fruits of the vector (pSa13)-transformed control, OE-wtBjHMGS1 (430 and 445) and OE-S359A (622 and 625). H, value higher than the control; L, value lower than the control. Values are means ± SD (n=3). a indicates significant difference between HMGS-OE and the vector (pSa13)-transformed control; b indicates significant difference between OE-wtBjHMGS1 and OE-S359A (*P*< 0.05, Student’s *t*-test).

**Table S1.** Oligonucleotide primers used in this study.

| Primer | Length | Sequence (5’-3’) | Location (nucleotide positions) | Orientation |
| --- | --- | --- | --- | --- |
| *35S* | 25-mer | CAATCCCACTATCCTTCGCAAGACC | 7372 to 7396 of *CaMV* genome (Franck et al. 1980) | Forward |
| ML264 | 30-mer | GGATCCATAACCAATGGACACTGAGGATCC | 1427 to 1444 in *BjHMGS1* mRNA (GeneBank accession No. AF148847)  Restriction site *Bam*HI underlined | Reverse |
| ML276 | 30-mer | GGATCCATGGCGAAGAACGTAGGGATATTG | 59 to 82 in *BjHMGS1* mRNA (GeneBank accession No. AF148847)  Restriction site *Bam*HI underlined | Forward |
| ML860 | 22-mer | GGAGACTGGTTCTCGCAGAGAC | 1164 to 1185 in *BjHMGS1* mRNA (GeneBank accession No. AF148847) | Reverse |
| ML915 | 20-mer | CATTGCTATGTTGATAGGAC | 499 to 518 in *BjHMGS1* mRNA (GeneBank accession No. AF148847) | Forward |
| ML1666 | 20-mer | TGGGCGTCTTGAAGTAGGCA | 225 to 244 in *BjHMGS1* mRNA (GeneBank accession No. AF148847) | Forward |
| ML1667 | 18-mer | AAAGCCGCAGTTCCACCA | 354 to 371 in *BjHMGS1* mRNA (GeneBank accession No. AF148847) | Reverse |
| ML1668 | 20-mer | CAATGCTGTGAGATGCCTGT | 730 to 749 in *SlHMGR1* mRNA (GeneBank accession No. L40938) | Forward |
| ML1669 | 21-mer | ACTCTCTCCCATCAAGCAACA | 788 to 808 in *SlHMGR1* mRNA (GeneBank accession No. L40938) | Reverse |
| ML1670 | 20-mer | GGCTGTAAATGATGGCAAGG | 1512 to 1531 in *SlHMGR2* mRNA (GeneBank accession No. M63642) | Forward |
| ML1671 | 20-mer | ATTTGACCCTGGTGCCTCTC | 1546 to 1665 in *SlHMGR2* mRNA (GeneBank accession No. M63642) | Reverse |
| ML1672 | 19-mer | TCACCCGAACCCAGACGAA | 492 to 510 in *SlIPI* mRNA (GeneBank accession No. EU253957) | Forward |
| ML1673 | 22-mer | TCCCACCATTTGAACAAGAAGT | 623 to 644 in *SlIPI* mRNA (GeneBank accession No. EU253957) | Reverse |
| ML1674 | 20-mer | TTCGCAACCACATCACCAGA | 374 to 393 in *SlFPPS1* mRNA (GeneBank accession No. AF048747) | Forward |
| ML1675 | 22-mer | CGGCGATGAATAGACAATGAGT | 533 to 554 in *SlFPPS1* mRNA (GeneBank accession No. AF048747) | Reverse |
| ML1676 | 20-mer | TCAGCAGATGCCGATGGTTA | 44 to 63 in *SlGPPS* mRNA (GeneBank accession No. DQ286930) | Forward |
| ML1677 | 20-mer | ATGGGTCCACTTGCTCCTCT | 213 to 232 in *SlGPPS* mRNA (GeneBank accession No. DQ286930) | Reverse |
| ML1678 | 22-mer | AGGAGGTGGAAACAACTGATGA | 470 to 491 in *SlSQS* mRNA (GeneBank accession No. GU075687) | Forward |
| ML1679 | 20-mer | AGAACATACGGCACTTGGGT | 663 to 682 in *SlSQS* mRNA (GeneBank accession No. GU075687) | Reverse |
| ML1680 | 18-mer | GCTCGCTTTGTGACCCTA | 777 to 794 in *SlSQE* mRNA (GeneBank accession No. AF167426) | Forward |
| ML1681 | 22-mer | TCCGTGCTACTGATAGGATAAA | 906 to 927 in *SlSQE* mRNA (GeneBank accession No. AF167426) | Reverse |
| ML1682 | 19-mer | CTTTGCTGCTGGGTTGAAG | 1123 to 1141 in *SlCAS1* mRNA (GeneBank accession No. EU449280) | Forward |
| ML1683 | 19-mer | TTCTTCGCCAAACCCTGTC | 1281 to 1299 in *SlCAS1* mRNA (GeneBank accession No. EU449280) | Reverse |
| ML1684 | 20-mer | GCAGGGCAGATTGTGGACTT | 673 to 692 in *SlGGPPS1* mRNA (GeneBank accession No. DQ267902) | Forward |
| ML1685 | 20-mer | CATTCGCTCCACATCAACCT | 809 to 828 in *SlGGPPS1* mRNA (GeneBank accession No. DQ267902) | Reverse |
| ML1686 | 20-mer | GGGATTGGAAAAGGCTAAGG | 960 to 979 in *SlGGPPS2* mRNA (GeneBank accession No. DQ267903) | Forward |
| ML1687 | 20-mer | AGCAATCAATGGAGCAGCTT | 1040 to 1059 in *SlGGPPS2* mRNA (GeneBank accession No. DQ267903) | Reverse |
| ML1688 | 24-mer | GGTATTGTGTTGGACTCTGGTGAT | 581 to 604 in *SlACTIN* mRNA (GeneBank accession No. BT013524) | Forward |
| ML1689 | 19-mer | ATTTCCCGTTCAGCAGTGG | 738 to 756 in *SlACTIN* mRNA (GeneBank accession No. BT013524 ) | Reverse |
| ML1690 | 21-mer | CAATCCCGCTTCGTCTACTTT | 527 to 547 in *CYP710A11* mRNA (GeneBank accession No. AB223043) | Forward |
| ML1691 | 19-mer | CTAATCTCCCAACTGCTAA | 706 to 724 in *CYP710A11* mRNA (GeneBank accession No. AB223043 ) | Reverse |
| ML1692 | 18-mer | GCGGATTTCAGGCAAGGA | 653 to 670 in *SlCYP85A1* mRNA (GeneBank accession No. U54770) | Forward |
| ML1693 | 21-mer | CATAGAAGTGGTGGAAACAGT | 838 to 858 in *SlCYP85A1* mRNA (GeneBank accession No. U54770) | Reverse |
| ML1694 | 21-mer | CCACATACTTGGTTGCCCTAC | 213 to 233 in *SlCYP85A3* mRNA (GeneBank accession No. AB190445) | Forward |
| ML1695 | 20-mer | ATGGACTGTGGGTAGCCTGG | 306 to 325 in *SlCYP85A3* mRNA (GeneBank accession No. AB190445) | Reverse |
| ML1696 | 20-mer | CGTCCCCTAAAGCATTCTCA | 752 to 771 in *TRR3/4* mRNA (Unigene SGN-U577676) | Forward |
| ML1697 | 20-mer | CGTCTTGTTGGTGATGTTGG | 829 to 848 in Sl*TRR3/4* mRNA (Unigene SGN-U577676) | Reverse |
| ML1698 | 20-mer | TGCTTAGAAGAAGGGGCAGA | 479 to 498 in *SlTRR8/9a* mRNA (Unigene SGN-U572841) | Forward |
| ML1699 | 20-mer | GGGGGCTTTTACATTTGGTT | 562 to 581 in *SlTRR8/9a* mRNA (Unigene SGN-U572841) | Reverse |
| ML1700 | 20-mer | AGTATGCCGGAAATGACTGG | 367 to 386 in *SlTRR8/9b* mRNA (Unigene SGN-U572839) | Forward |
| ML1701 | 20-mer | TGGAACATTTTCCGATGACA | 449 to 468 in *SlTRR8/9b* mRNA (Unigene SGN-U572839) | Reverse |
| ML1702 | 20-mer | GGTCTAAGGGCGTTGGAGTA | 216 to 235 in *SlTRR16/17* mRNA (Unigene SGN-U601012) | Forward |
| ML1703 | 20-mer | TCCTGGCATGCAATAATCTG | 304 to 323 in *SlTRR16/17* mRNA (Unigene SGN-U601012) | Reverse |
| ML2332 | 20-mer | AACCTTTGCTGCTGGATTGG | 1359 to 1378 in *SlDXS1* mRNA (GeneBank accession No. FN424051) | Forward |
| ML2333 | 22-mer | ATCAACGTCATGCACTACCTGG | 1440 to 1461 in *SlDXS1* mRNA (GeneBank accession No. FN424051) | Reverse |
| ML2334 | 23-mer | AGCAGGCTTAGCTACAGAAGGTC | 1284 to 1306 in *SlDXS2* mRNA (GeneBank accession No. FN424052) | Forward |
| ML2335 | 20-mer | CCGTCTGCACCTACCAAACC | 1417 to 1436 in *SlDXS2* mRNA (GeneBank accession No. FN424052) | Reverse |
| ML2336 | 25-mer | CTTAGGCGCATTATATTAACTGCAT | 733 to 757 in *SlDXR* mRNA (GeneBank accession No. AF331705) | Forward |
| ML2337 | 25-mer | GTGGCAGAATCAACAGTAATCTTTT | 848 to 872 in *SlDXR* mRNA (GeneBank accession No. AF331705) | Reverse |
| ML2338 | 20-mer | GGATCGAGTCCTCCGGGTTA | 712 to 731 in *SlGPPS-SSU-II* mRNA (GeneBank accession No. AK325077) | Forward |
| ML2339 | 20-mer | AGCATTTGGTCCACCCTCAA | 792 to 811 in *SlGPPS-SSU-II* mRNA (GeneBank accession No. AK325077) | Reverse |
| ML2340 | 20-mer | GGCCATTTGACATGCTCGAT | 572 to 591 in *SlPSY–1* mRNA (GeneBank accession No. EF534739) | Forward |
| ML2341 | 22-mer | TCCATACGCATTCCTTCAATCA | 650 to 671 in *SlPSY–1* mRNA (GeneBank accession No. EF534739) | Reverse |
| ML2342 | 21-mer | CGGCCATTTGATATGCTTGAT | 643 to 663 in *SlPSY–2* mRNA (GeneBank accession No. EF534738) | Forward |
| ML2343 | 21-mer | CCATACGCATTCCTTCAACCA | 722 to 742 in *SlPSY–2* mRNA (GeneBank accession No. EF534738) | Reverse |
| ML2344 | 20-mer | CAGGAGAATTCAGCCGCTTT | 608 to 627 in *SlPDS* mRNA (GeneBank accession No. NM_001247166) | Forward |
| ML2345 | 20-mer | TCCGGCCATGTAAGCATTTC | 688 to 702 in *SlPDS* mRNA (GeneBank accession No. NM_001247166) | Reverse |
| ML2346 | 19-mer | TTGGAGCGTTCGAGGCAAT | 1219 to 1237 in *SlZDS* mRNA (GeneBank accession No. AF195507) | Forward |
| ML2347 | 24-mer | AGAAATCTGCATCTGGCGTATAGA | 1268 to 1291 in *SlZDS* mRNA (GeneBank accession No. AF195507) | Reverse |
| ML2348 | 22-mer | CATTTTGGCGGAATCAACTACC | 951 to 972 in *SlCRTISO* mRNA (GeneBank accession No. AF416727) | Forward |
| ML2349 | 23-mer | CATAAAACTTCCTCCCGTCAGAA | 1139 to 1161 in *SlCRTISO* mRNA (GeneBank accession No. AF416727) | Reverse |
| ML2350 | 22-mer | CTTTTCTAAGTCCCACCACCAA | 83 to 104 in *SlLCY-B* mRNA (GeneBank accession No. AF254793) | Forward |
| ML2351 | 21-mer | ACTCTGGCTTTGATGTGGGTG | 173 to 193 in *SlLCY-B* mRNA (GeneBank accession No. AF254793) | Reverse |
| ML2352 | 23-mer | AATGTTATGGCTATTGGTGGGAA | 1042 to 1064 in *SlCYC-B* mRNA (GeneBank accession No. Y18297) | Forward |
| ML2353 | 25-mer | GACCCTCTTATCATTCTTGTTGAGC | 1157 to 1181 in *SlCYC-B* mRNA (GeneBank accession No. Y18297) | Reverse |
| ML2354 | 22-mer | CATCCAGCCACAGGTTATTCAG | 1144 to 1165 in *SlLCY-E* mRNA (GeneBank accession No. Y14387) | Forward |
| ML2355 | 23-mer | TCGGGATACTTGAACTGGTAAGC | 1245 to 1267 in *SlLCY-E* mRNA (GeneBank accession No. Y14387) | Reverse |
| ML2356 | 22-mer | AAGACTGAGAGCCGATTCCAAA | 873 to 894 in *SlGGPPR* mRNA (Unigene SGN-U564570) | Forward |
| ML2357 | 21-mer | CCCCAACTAATGCGACTCTGT | 968 to 988 in *SlGGPPR* mRNA (Unigene SGN-U564570) | Reverse |
| ML2358 | 24-mer | GATGCTCAGGGATTAGGTGATAAG | 681 to 704 in *SlGMTT* mRNA (Unigene SGN-584511) | Forward |
| ML2359 | 20-mer | ATGATTGTGCCTCCTGGTGC | 837 to 856 in *SlGMTT* mRNA (Unigene SGN-584511) | Reverse |
| ML2360 | 25-mer | ACATAGGGATGATGGTGGTCACCTT | 20 to 44 in *SlMTS1* mRNA (TC166486) | Forward |
| ML2361 | 22-mer | CTGAACGCCTTGTGGTGGAAAT | 121 to 142 in *SlMTS1* mRNA (TC166486) | Reverse |
| ML2362 | 22-mer | CCTCTCCACTGGACAGCCACTT | 15 to 36 in *SlMTS1-like* mRNA (GeneBank accession No. AW617523) | Forward |
| ML2363 | 23-mer | CCACATGGTAGGCTCGTAATTCC | 110 to 132 in *SlMTS1-like* mRNA (GeneBank accession No. AW617523) | Reverse |
| ML2364 | 25-mer | AGCAAACCTTAGAACAAACAAGCAA | 4 to 28 in *SSTLE1* mRNA (TC162858) | Forward |
| ML2365 | 24-mer | CCAAACAGATGGGTGAAAATTAGC | 63 to 86 in *SSTLE1* mRNA (TC162858) | Reverse |
| ML2366 | 20-mer | CGAATTCGGCACGAGCTTCT | 8 to 27 in *SSTLE2* mRNA (GeneBank accession No. AF279454) | Forward |
| ML2367 | 20-mer | TCGTTGCATCGCGTCTATCA | 200 to 219 in *SSTLE2* mRNA (GeneBank accession No. AF279454) | Reverse |
| ML2368 | 25-mer | GAACTCATCAACACAATCCAATGTC | 274 to 298 in *SSTLH3* mRNA (GeneBank accession No. AW616373) | Forward |
| ML2369 | 24-mer | ATAGCATGAAGATCACCAATCGAA | 372 to 395 in *SSTLH3* mRNA (GeneBank accession No. AW616373) | Reverse |
| ML2370 | 33-mer | GGTTCTTCAATGTTGGAAATTATCATCTCCATC | 12-44 in *SlCPT1* mRNA (GeneBank accession No. NM_001247704) | Forward |
| ML2371 | 25-mer | GCAGACACTGTCAGAGGCGAATTTG | 113 to 137 in *SlCPT1* mRNA (GeneBank accession No. NM_001247704) | Reverse |
| ML2372 | 27-mer | GATCTTCAATGCTGGAAATCATCATCA | 55 to 81 in *SlCPT2* mRNA (GeneBank accession No. JX943884) | Forward |
| ML2373 | 27-mer | AAGCTCTTCATGAAGCTCGAGATCATC | 235 to 261 in *SlCPT2* mRNA (GeneBank accession No. JX943884) | Reverse |
| ML2374 | 20-mer | GGACAAGAATGAGCACCGGA | 615 to 634 in *SlCPT3* mRNA (GeneBank accession No. JX943885) | Forward |
| ML2375 | 21-mer | ATCTCAGGCCATAGTGCAGCA | 771 to 791 in *SlCPT3* mRNA (GeneBank accession No. JX943885) | Reverse |
| ML2376 | 21-mer | GAATCGTTCCGAGGAGGTTGA | 342 to 362 in *SlCPT6* mRNA (GeneBank accession No. JX943888) | Forward |
| ML2377 | 26-mer | ATTTTGTCGGAAGTTTTGATTTACCT | 435 to 460 in *SlCPT6* mRNA (GeneBank accession No. JX943888) | Reverse |
